# Privileged interests on the party agenda: Bitcoin-related issues in two countries since 2020

**DOI:** 10.1057/s41269-024-00380-4

**Published:** 2024-12-23

**Authors:** Joost van Spanje, Costin Ciobanu, Greta Arancia Sanna

**Affiliations:** 1 Royal Holloway, University of London, London, United Kingdom; 2 Department of Political Science, Aarhus University, Bartholins Allé 7, 8000 Aarhus, Denmark

## Abstract

What explains whether and how much political parties give attention to a policy issue? Parties are expected to cater to the interests of privileged groups and to be dismissive unless an issue offers an electoral opportunity. How to test this? Most issues have been around for so long that it has become difficult to track party responses. Ideally, a multifaceted phenomenon would fall from the sky and become favored by privileged groups, after which we would observe party reactions. Bitcoin fulfills these criteria. It has become significant, suited for various ideologies yet disproportionally supported by wealthy, highly educated male voters. In this paper, we study how new issues emerge around Bitcoin, and how parties respond. Voter attitudes, preferences, expectations, and Bitcoin ownership are taken from our 2020-2023 four-wave British (*N*=5,121) and Dutch (*N*=5,002) voter surveys. Party positions on Bitcoin are derived from our surveys of MPs and party representatives, cross-validated by party communication. Using issue yield theory, we find that weak electoral incentives, particularly due to age-related internal divisions, prevent parties from catering to privileged groups on Bitcoin issues. This suggests that a party system will not embrace elite interests, even on low-salience issues, under unfavorable public opinion alignments.

**Supplementary Information:**

The online version contains supplementary material available at 10.1057/s41269-024-00380-4.

## Introduction

“Why does Bitcoin make Elizabeth Warren toss and turn and twitch at night? Because she wants her sticky little socialist fingers to be able to control every penny in every one of our bank accounts.” Ted Cruz lambasted his fellow US Senator for her scepticism of Bitcoin. Together with the Texan Governor, the Republican lawmaker aims to turn his state into an “oasis on planet Earth for Bitcoin and other crypto.”^1^ Similarly, the mayors of Miami, Austin, Tampa, and New York City all intend to create a “crypto hub.” Other pro-Bitcoin politicians included several 2020 and 2024 presidential candidates.

Bitcoin also entered presidential campaigns in Argentina and South Korea. In Canada, the Opposition Leader sees Bitcoin as an inflation hedge – to which the Prime Minister responded, “Telling people they can opt out of inflation by investing in cryptocurrencies is not responsible leadership.”^2^ Meanwhile, the (now former) British Prime Minister positioned the country as a “global hub” for the crypto industry, Bitcoin has been made legal tender in El Salvador, and Bhutan as well as Oman is investing in crypto mining. At the city level, aspiring crypto hubs include Hong Kong, Rio de Janeiro, Busan, Lugano, and Singapore.

These examples are exceptions to the general rule of politician silence about Bitcoin. Silence is arguably their default reaction to any new topic. Why do some politicians break this silence and take a stance – and why pro or con? Generally, political actors cannot position themselves on every issue. Actually, they may not have an interest in doing so. Strategic actors ignore issues that present risks while taking up positions that offer opportunities, especially electoral ones. Furthermore, the vast literature on unequal representation shows that parties tend to emphasize issues that reflect the concerns of educated, affluent male citizens (for a review of the literature, see Elsässser and Schäfer 2023; for work directly related to our argument, see Weber 2020).

It is not easy to model and explain which positions parties take up. Most policy issues have already been around for decades, which makes it difficult to tease out responses by a party, reactions to these responses, input from other stakeholders, repositioning of a party, effects of news media coverage of all this, and so on. Ideally, a genuinely new phenomenon would be introduced, and we would track political behavior. That phenomenon must gain traction for parties to react. More ideally, it would have a clear starting point. Even more ideally, it would be a truly exogenous rarity. More ideally still, it would be a prodigy that is multifaceted, so that it allows for voters to develop various types of views on it while different parties can easily link it to their ideologies. And again even more ideally, the phenomenon would be supported by a clearly privileged group.

Bitcoin is a phenomenon that ticks all these boxes. Invented by a person or group that went under the name of Satoshi Nakamoto, it has little in common with anything that existed before.^3^ Since its starting date of 3 January 2009, Bitcoin has become significant in many respects. This has forced some political actors to respond and will likely elicit more political responses. It is also multifarious, so that it lends itself to being linked to various ideologies, providing arguments for all kinds of political actors to reject or accommodate it. For parties, it has simultaneously opened up a range of options, each associated with a mix of risk and opportunity. In other words, we have witnessed the birth of a fresh set of issues. Furthermore, a group of wealthy high-educated male citizens is positive about Bitcoin and often owns it. This allows us to study how political actors respond to emerging issues that are suddenly brought to the table and cater to the interests of these groups. In this paper, we do so in two countries with developed financial systems (the UK and Netherlands) since 2020. Using party positions on Bitcoin and nationally representative surveys of voters, we provide a first descriptive assessment of whether parties in these two countries have an electoral incentive to talk about crypto and to engage in unequal representation on behalf of these traditionally privileged groups.

## Relevant prior studies on Bitcoin

Bitcoin has not only received attention from a few high-profile politicians, but also from scholars. A Google Scholar search using “Bitcoin” as the keyword returns about 350,000 hits. However, the bulk of these studies is unrelated to politics. While several books link it to ideology (Golumbia 2016; Tseng et al. 2022) or religion (Breedlove et al. 2020) or discuss various future scenarios (Warren 2023), Bitcoin and politics have never been empirically analyzed, as far as we know. Whereas its price fluctuations have been explored in depth,^4^ its adoption in politics has yet to be rigorously examined.

Relevant areas of research include those of Bitcoin users and political narratives associated with Bitcoin. About Bitcoin users, Bohr and Masooda (2014) employ publicly available survey data of Bitcoin users to explore the structure of the Bitcoin community, including wealth, optimism about the future of Bitcoin and themes attracting users to cryptocurrency. Here, results suggest that variables such as young age, spending Bitcoin on illicit goods and participating in Bitcoin specific forums positively predict Bitcoin accumulation. The study also presents ideological explanations of the differentiated user attraction to Bitcoin. Where left-wing users might be attracted to Bitcoin as a decentralized payment system that challenges power structures, libertarians view Bitcoin as an alternative currency that has the capacity to free the individual from state power structures. When it comes to the political narratives associated with Bitcoin, Golumbia (2016) argues that the problems that Bitcoin advocates consider central are not those relating to money or currency but are instead largely ideological. These include the desire to bypass central banks and credit card blockades for the creation of money or the provision of financial services. These ideas, Golumbia (2016) argues, emerge from ideological and conspiratorial anti-central bank rhetoric that originates from the US extreme right. This said, positions favorable to Bitcoin are not restricted to the extreme right, as we will see later on in this paper when we map the positions of 32 parties in the two countries.

## Theoretical considerations

Our theoretical reasoning starts from Issue Yield Theory. Developed by De Sio and Weber (2014), this theory leverages the multidimensional nature of party competition. It argues that parties will seek to maximize their public support by focusing on issues that unite the party base, while also attracting support from the larger electorate (see a similar argument by Hobolt and de Vries 2015). Issue yield is defined as “the degree to which an issue allows a party to overcome the conflict between protection and expansion of electoral support” (871). The theory seeks to predict, from basic information about voter distributions, the policies that the parties will emphasize in their quest for votes. Specifically, the issue yield for a specific issue*party combination can be calculated by looking only at the proportion of the electorate supporting a policy (*i*), the proportion of the electorate supporting a party (*p*), and the proportion of the electorate supporting both policy and party (*f*) (873). The main prediction of this theory is that “issue yield has a positive and significant effect on issue emphasis” (879). As it has already been used to explain electoral performance (De Sio and Weber 2020) or how the parties reacted to the 2008 financial crisis (De Sio et al. (2016), we will employ this general model of party competition to understand whether and how it applies to new issues that Bitcoin has spawned. Here, parties are expected to discuss Bitcoin more frequently when it has a high absolute issue yield and to speak positively about it when it has a positive yield.

Within this framework, we focus on the literature on how party agendas include issues. Specifically, we consider Weber’s (2020) argument that party agendas embrace the interests of privileged groups. According to Weber (2020), potential electoral backlash is anticipated and fended off by parties through a strategy of “discreet” inequality, whereby parties build their policy agendas by inconspicuously emphasizing some issues and de-emphasizing others to cater to privileged constituencies.

This strategy aligns with the considerable evidence of unequal representation in our political systems. As Elsässer and Schäfer (2023, 469) conclude in their review of the literature, “not only are the opinions of decision-makers more congruent with those of the better off, but policy choices also reflect their preferences more clearly.” Specifically, Gilens (2005) shows that, in the US, policy outcomes reflect the preferences of the most affluent. The same responsiveness to the opinions of the rich is also observed in Europe (Elsässer et al. 2018; Schakel 2021). Furthermore, research finds that the preferences of the higher educated are more reflected in government decisions (Rosset and Stecker 2019; Schakel and Van Der Pas 2021). Unequal responsiveness is not limited to income and education, but also has a gender dimension: with data from 12 European countries, Homola (2019, 958) finds that parties are more responsive to policy preference shifts among men than among women. Thus, in general, parties seem to favor the more privileged segments of society – mainly wealthier, more highly educated men.

Recall that wealthy well-educated male citizens tend to own Bitcoin at higher rates, and hold it in high regard more generally. What would one then, a priori, expect from party positions on Bitcoin issues? Prior studies suggest that, given that these are issues that privileged groups tend to care about, parties in general will tend to pay attention to these issues (Weber 2020). This is because, for some reason, “public policy favors socioeconomic elites” as, among other things, “parties pay unequal attention to different subconstituencies when assembling their campaign platforms” (1768). Thus, one would expect that, all things being equal, *parties will put Bitcoin on their agenda to cater to the interests of educated, affluent male citizens*, as this group has a disproportionate interest in Bitcoin and can be considered traditionally privileged, as per Weber (2020, 1772).

Going beyond this general expectation, we build on Weber’s (2020) point that we could gain more insights into unequal representation by looking at two aspects: (1) the distribution of voter preferences on the issue; and (2) different types of parties.

First, the distribution of voter preferences on the issue could be relevant in understanding whether we should expect unequal responsiveness or equal treatment by parties regarding Bitcoin. Weber (2020) argues that bias in representation will be small if: (1) voter views differ markedly along sociodemographic lines, meaning that unresponsiveness “decreases with the degree to which policy support differs between a privileged group and its marginalized counterpart” (Weber 2020, 1773); and (2) support for an issue is higher among a traditionally privileged group than among the traditionally marginalized group (Weber 2020, 1773). If there are differences among social groups regarding the issue, and Bitcoin is viewed considerably more favorably by the privileged groups (two aspects that will be empirically investigated), then we would *not* expect significant discrimination in party responsiveness between groups on the issues associated with Bitcoin.

Second, some parties traditionally target privileged groups. Given that these groups are, as we will later show, favorable to Bitcoin, they could put this issue on the agenda in a positive way, as this could be beneficial to them electorally. Concretely, we would expect parties to react to Bitcoin in accordance with their traditional alignments (Weber 2020, 1790). Given its traditional alignment with privileged groups, it is likely that *the economic right will rally in favor of Bitcoin*. Similarly, other parties aim to accommodate marginalized groups. They thus mobilize on issues that serve this constituency, sometimes by going against policies preferred by privileged groups. So, we could expect that *the economic left*, that attracts support from those who may not understand crypto and who risk losing money, *will mobilize against Bitcoin*. Finally, for other parties, the incentives are not strong enough to override the urge to avoid a “negative vote” (Weber 2020). For them, talking about Bitcoin does not make sense strategically and electorally. Hence, we expect that all other parties will not mention Bitcoin, to avoid upsetting the few voters who care about it.

## Data and methods

We test our expectations based on two types of data sets that we have compiled for this purpose, party surveys and voter surveys. The one gives us party positions, the other offers us voter positions. We conduct our study in the UK and Netherlands, two countries with developed financial systems. Historically, they have strong trade and banking traditions and host arguably Europe’s largest financial centers. These countries have perhaps the world’s best developed banking and payment systems in a setting with low political and financial repression. This means that citizens in these countries do not look to Bitcoin for making payments (as in countries with less developed payment systems), for making pseudonymous financial transactions such as political donations (as is the case under dictatorial rule) or for circumventing capital controls, sending remittances or fighting inflation (as in many developing countries). British and Dutch citizens who like Bitcoin typically do so for financial speculation or ideological reasons, not because they directly need it.

First, for the supply side of the electoral market, we measured the position vis-à-vis Bitcoin for each party in each of the two countries. For this, from May through September 2023 we contacted each of the parties to ask its position. See Appendix B for the list of parties, and Appendix C for their answers. Out of 32 parties, 21 responded. To complement and cross-validate their replies to us, we added information from other sources, including every mentioning of Bitcoin in a full-text database of parliamentary speeches in both countries (Sylvester et al. 2022), see Appendix D. For the UK case, a question was included in a questionnaire of a standard survey of MPs fielded by YouGov in July 2023. That question asked their party’s attitude toward Bitcoin on a scale from −5 (“fully avoid all risks, e.g., by a ban”) to +5 (“fully seize all opportunities”) with a “do not know” option as well as the option “party does not have a position.” A party that advocates an outright (de facto) ban of Bitcoin is coded “−5,” whereas a party that is completely uncritically and unconditionally in favor is coded “+5.” The median of party answers was included for each party, unless a majority indicated that the party had no stance. A total of 108 MPs from eight parties reacted. For the Dutch parties, we looked up their answers in two prior surveys. This results in data points for each party with regard to the extent to which, and how, it takes a position on Bitcoin. The Dutch parties were placed on the same scale, ranging from −5 to +5. The inter-coder reliability was high.^5^ To give a sense of the robustness of our findings, we obtain similar results using various operationalizations of the party positions.

Second, for the demand side, we have carried out two four-wave cross-sectional surveys simultaneously, one in the United Kingdom and the other in the Netherlands between 2020 and 2023.^6^ Voters have indicated their vote intention as well as a range of perceptions, attitudes, interests, preferences, and expectations regarding Bitcoin. From the positions of party supporters and from the electorate in its entirety, we calculate Bitcoin issue yield for each party, but also for the privileged groups discussed by Weber (2020): men, the highly educated, and the affluent.

As the surveys contain a multitude of items related to Bitcoin, we group them into four indices that are common for both countries and are based on the same items.^7^ These four indices are the following: one pertaining, in our interpretation, to positive aspects, effects, and expectations regarding Bitcoin, one pertaining to an idea of Bitcoin as a valueless coin that has various negative effects, one referring to support for punitive governmental action against crypto, and, finally, one pertaining to past, current or expected crypto ownership. For each country and each index, we additively aggregate the corresponding items by summing up the items and taking the mean. Following De Sio and Weber (2014), we dichotomize the support for each index by country, based on the midpoint of the scale. In the UK data (*N* = 5,121), 15% have a positive notion of Bitcoin, 33% hold a negative view, 28% are in support of restrictive governmental intervention, while 15% report past, current, or expected crypto ownership. In the Dutch data (*N *= 5,003), only 3% acknowledge a positive impact of Bitcoin, 30% see it as a worthless evil, 33% favor punitive governmental action, and 7% express past, current, or expected crypto ownership. These levels of support of different crypto issues allow us to identify the proportion of the electorate supporting the policy (*i*), a first component in calculating issue yield.

The second component key in obtaining issue yield is the proportion of the electorate supporting the party (*p*). For the UK, this is obtained based on the respondents’ reported vote in the 2019 General Election. For the Dutch sample, the electoral support is derived from the vote intention in a future General Election (first three waves) and reported vote in the 2021 General Election (wave 4). Next, we calculate, for each issue, the proportion of the electorate supporting both policy and party (*f*).

Finally, we calculate the scaled issue yield by applying the formula proposed by De Sio and Weber (2014, 877): *Scaled issue yield* $= \frac{f - ip}{p(1 - p)} + \frac{i - p}{1 - p}$. In our datasets, we have information on 11 British and 21 Dutch parties.

We analyze the Bitcoin-related party – voter connection in three ways. First, we assess whether there is a critical mass of crypto holders among the support base of a particular party. Second, to evaluate the potential for unequal representation, we do two things: we look at the socio-demographic determinants of crypto ownership to investigate whether the better-off are more likely to own Bitcoin; then, we analyze whether the privileged are more likely to hold a positive view of Bitcoin. Third, for each party we analyze the relationship between issue yield, on the one hand, and positioning, on the other. We do so for the whole electorate but also for men, the well-educated, and the well-off. This provides insights into whether the parties’ positions on Bitcoin align with the issue’s electoral potential among all voters or only among privileged groups.

## Findings

Looking at the demand side of the electoral market,^8^ we know that citizens can, wittingly or unwittingly, base their opinion on narrow material interest (such as owning Bitcoin) or their policy or ideological convictions (such as being critical of government intervention in the economy), or a combination of these two.

**Figure 1:**
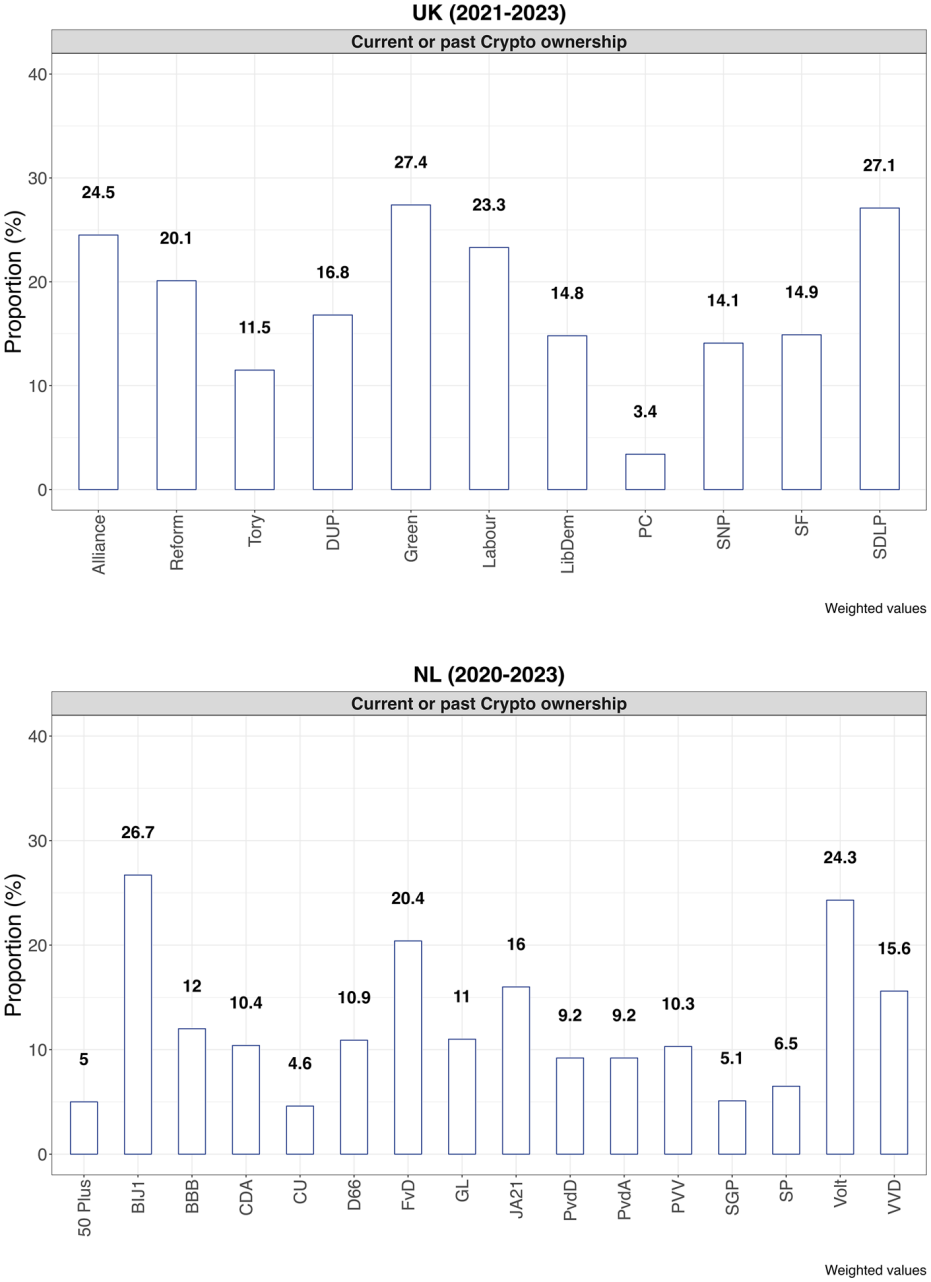
Crypto ownership by party in the UK and the Netherlands, 2020–2023

Let us first look at Bitcoin ownership.^9^ Figure 1 presents the distribution of current and past crypto ownership by party support.

In the UK, we see that 27.4% of Green Party supporters have or have had crypto holdings, just as 27.1% of SDLP voters, and 24.5% of Alliance voters. Interestingly, none of these parties has a position on Bitcoin at all for the time being. Similarly, of the top three Dutch parties whose supporters own or have owned crypto, only FvD (20.4% ownership) openly backs Bitcoin, while BIJ1 (26.7%) has a critical stance without publicizing it, and Volt (24.3%) does not yet have a position. Thus, in both countries, we see significant variation in terms of the share of supporters who have ever possessed crypto. At the same time, we get some *prima facie* indication that the parties’ positions on Bitcoin are not aligned with the partisan supporters’ crypto holdings.

Five out of six of the joint top three of either country are progressive parties, the sixth one (FvD) is far right. Unlike FvD, the five progressive parties are cross-pressured by an increasing share of their supporters having environmental worries, as we will discuss below.

Who are these voters who have purchased crypto? Let us see to what degree socio-demographic and political variables explain current or past crypto ownership. We start in the United Kingdom, see Table 1 (model 1).

**Table 1:** Determinants of currently and ever owning crypto in the UK and the Netherlands, 2020–2023

	UK		Netherlands	
	Currently owning	Ever owning	Currently owning	Ever owning
	(1)	(2)	(3)	(4)
Female	$$-0.08 \; (0.01)^{***}$$	$$-0.10 \; (0.01)^{***}$$	$$-0.07 \; (0.01)^{***}$$	$$-0.10 \; (0.01)^{***}$$
Age under 40	$$0.15 \; (0.01)^{***}$$	$$0.22 \; (0.01)^{***}$$	$$0.05 \; (0.01)^{***}$$	$$0.06 \; (0.01)^{***}$$
Age over 60	$$-0.06 \; (0.01)^{***}$$	$$-0.11 \; (0.01)^{***}$$	$$-0.06 \; (0.01)^{***}$$	$$-0.09 \; (0.01)^{***}$$
University education	$$-0.01 \; (0.01)$$	$$-0.03 \; (0.01)^{**}$$	–	–
Income > £40k	$$0.02 \; (0.01)^{***}$$	$$0.02 \; (0.01)^{*}$$	–	–
Savings > £10k	$$0.02 \; (0.01)^{**}$$	$$0.01 \; (0.01)$$	–	–
Real estate > £10k	$$0.05 \; (0.01)^{***}$$	$$0.06 \; (0.02)^{***}$$	–	–
London residence	$$0.06 \; (0.01)^{***}$$	$$0.11 \; (0.02)^{***}$$	–	–
Conservative vote (2019)	$$-0.03 \; (0.01)^{***}$$	$$-0.04 \; (0.01)^{***}$$	–	–
Leave vote (2016)	$0.02 \; (0.01)^{*}$	$0.00 \; (0.01)$	–	–
Employee	–	–	$$-0.02 \; (0.01)^{**}$$	$$-0.02 \; (0.01)^{**}$$
High social class	–	–	$$0.04 \; (0.01)^{***}$$	$$0.05 \; (0.01)^{***}$$
Amsterdam	–	–	$-0.02 \; (0.02)$	$-0.01 \; (0.02)$
Income > €44k	–	–	$0.01 \; (0.01)$	$0.01 \; (0.01)$
Savings > €10k	–	–	$0.01 \; (0.01)$	$0.00 \; (0.01)$
Real estate > €10k	–	–	$$0.04 \; (0.01)^{***}$$	$$0.07 \; (0.01)^{***}$$
VVD supporter	–	–	$0.02 \; (0.01)^{*}$	$0.02 \; (0.01)$
Wave 2	$$0.03 \; (0.01)^{**}$$	$$0.01 \; (0.01)$$	$$0.05 \; (0.01)^{***}$$	$$0.05 \; (0.01)^{***}$$
Wave 3	$$0.03 \; (0.01)^{**}$$	$$0.03 \; (0.01)^{**}$$	$$0.05 \; (0.01)^{***}$$	$$0.06 \; (0.01)^{***}$$
Wave 4	$$0.05 \; (0.01)^{***}$$	$$0.08 \; (0.01)^{***}$$	$$0.06 \; (0.01)^{***}$$	$$0.07 \; (0.01)^{***}$$
Intercept	$$0.08 \; (0.01)^{***}$$	$$0.15 \; (0.02)^{***}$$	$$0.05 \; (0.01)^{***}$$	$$0.09 \; (0.02)^{***}$$
Adj. $\hbox {R}^2$	0.10	0.16	0.07	0.09
Observations	5121	5121	4991	4991

Note: $$^{***}p<0.01$$, $$^{**}p<0.05$$, $$^*p<0.1$$. OLS estimations. Standard errors shown in parentheses. Dependent variable for the models: currently owning crypto (binary variable) - models 1 and 3; ever owning crypto (binary variable) - model 2 and 4. All explanatory variables are binary. All estimations employ survey weights. Wave 1 is the reference category for the survey wave variable. The first two models use UK data, the last two Dutch data

Table 1 (model 1) shows significant differences between groups of respondents. On the one hand, we see that the elderly, women, and 2019 Tory voters are less likely to own crypto. On the other hand, the young, Londoners, high-income, the wealthy, homeowners, and Leave voters are more likely to do so.^10^ The findings for ever owning crypto are similar (model 2). Thus, in the UK, we have some evidence that crypto ownership is more likely among the privileged groups (men and the better-off financially); at the same time, the better educated are not more likely to own crypto.

We conduct the same estimations for the Dutch case, see Table 1 (model 3). Here, we find that the elderly, women, and employees are less likely to own crypto. At the same time, more likely to hold crypto are the young, the high class, homeowners, and VVD supporters. Again, the results for having ever possessed crypto look quite the same (model 4). In the Dutch case, we see that two privileged groups (the men and those with high social class) are more likely to own crypto, but this finding is not present for those with higher incomes.

For parties, these findings mean at least two things. First, crypto possession varies considerably from group to group, so that a party that targets, say, elderly females will likely remain shielded from the uncertainties Bitcoin may bring. We have substantial evidence that crypto is embraced by traditionally privileged groups, starting with men. Second, crypto adoption will likely continue to increase, for at least three reasons. One reason is generational replacement. In the long run, the young are bound to replace the elderly, who are far less likely to own crypto. A second reason is general growth. Table 1 also shows that, holding all else constant, the likelihood of owning crypto substantially increases over time in both countries. As a third reason, technological adoption often trickles down from the higher classes, the wealthy, and the cosmopolitan elites to others. This is what we seem to witness here as well as, for instance, well-off citizens and London residents were already holding crypto at high rates in the first wave. Taken together, these three trends suggest that – barring some unexpected event – parties must anticipate continued rapid growth in crypto adoption.^11^

**Figure 2:**
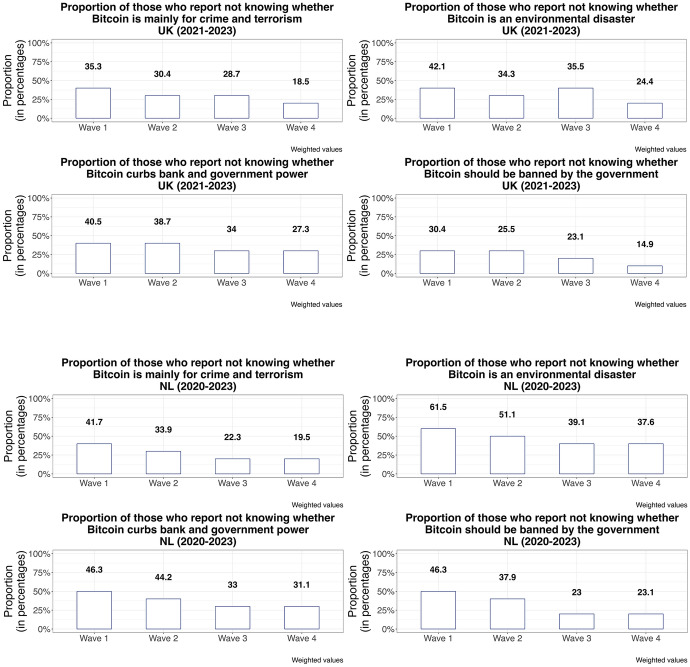
Share of respondents without opinion, various issues, the UK and the Netherlands, 2020–2023

When focusing on opinions, perceptions, and expectations, what is clear from the data is that voters gradually make their mind up about Bitcoin. We can tell from the shares of respondents who answer to various questions that they do not know. See Figure 2.

**Figure 3:**
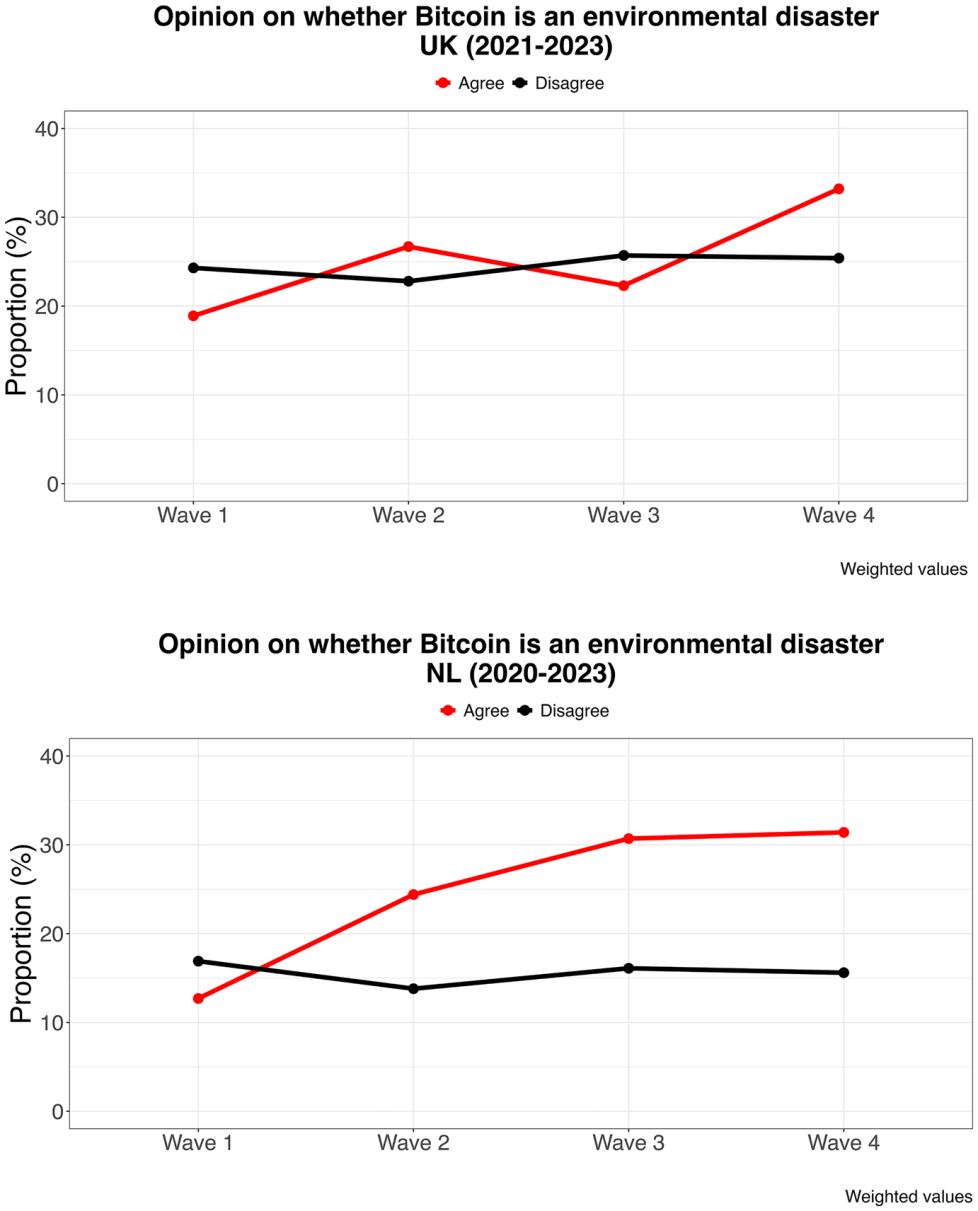
Voters’ opinion on Bitcoin’s environmental impact, the Netherlands and the UK, 2020–2023

Figure 2 makes clear that citizens are slowly forming opinions about Bitcoin. This increases polarization, as some citizens come to a positive conclusion about Bitcoin and others take a critical perspective. Again, this is an indication that we are dealing with a set of newly emerging policy issues that may or may not surge further and become politicized. An important issue threatening Bitcoin’s popularity is its environmental impact related to its proof-of-work consensus mechanism. Out of issues we asked about, environmental concerns show the clearest trend over time. See Figure 3.

The learning process described above works against Bitcoin on this topic, as more voters who make up their mind about it fall into the critical camp than into the favorable camp. In the UK, agreement with the thesis that Bitcoin presents an environmental disaster grew from 19 to 33%, and among Dutch voters even from 12% to 32% while disagreement remained fairly stable. The over-time increase in crypto ownership (see Appendix G), the decrease of those who do not have an opinion on the issue, and the variation in ownership among various parties suggest that Bitcoin could be perceived as a socially (and potentially politically) relevant phenomenon.

These trends likely put center and left parties under pressure, as they increasingly host both Bitcoin-friendly supporters, on the one hand, and supporters who are concerned about its environmental impact, on the other. In other words, Bitcoin may give rise to a “wedge issue” (van de Wardt et al. 2014). As a result, these parties will refrain from taking any position. This said, if they convince their supporters that Bitcoin can assist in the energy transition and help fight climate change, as has been pointed out,^12^ progressive parties can actually win votes on the Bitcoin ticket.

Meanwhile, parties at the right end of the political spectrum will be largely unaffected by these climate change worries. This is because, at least for now, few of their voters consider such environmental concerns salient: In the UK, the share of citizens who find environmental issues more generally extremely important stands at 20% among Reform UK supporters, whereas it reaches 40% among Labor voters, 46% among LibDems, and 50% among Greens; For Dutch voters we see a similar distribution, as PVV (6%), FvD (2%), and JA21 (0%) are at the bottom while PvdA (17%), GL (35%), and PvdD (38%) all score considerably higher.^13^

On the other hand, the favorable narrative about Bitcoin represented in our first index continues to hold up as well. Who buys this narrative, and who does not? Table 2 shows the results of regression analysis explaining support for this positive Bitcoin opinion.

**Table 2:** Determinants of positive Bitcoin impact views in the UK and the Netherlands, 2020–2023

	UK	Netherlands
	Bitcoin	Bitcoin
	positive impact	positive impact
	(1)	(2)
Female	$$-0.47 \; (0.04)^{***}$$	$$-0.37 \; (0.03)^{***}$$
Age under 40	$$1.23 \; (0.05)^{***}$$	$$0.18 \; (0.04)^{***}$$
Age over 60	$$-0.67 \; (0.06)^{***}$$	$$-0.34 \; (0.05)^{***}$$
University education	$-0.06 \; (0.05)$	–
Income > £40k	$-0.05 \; (0.05)$	–
Savings > £10k	$$-0.12 \; (0.05)^{**}$$	–
Real estate > £10k	$$0.22 \; (0.07)^{***}$$	–
London residence	$$0.39 \; (0.06)^{***}$$	–
Conservative vote (2019)	$0.04 \; (0.05)$	–-
Leave vote (2016)	$0.08 \; (0.05)$	–
Employee	–	$-0.03 \; (0.04)$
High social class	–	$$0.25 \; (0.04)^{***}$$
Amsterdam	–	$0.12 \; (0.09)$
Income > €44k	–	$0.02 \; (0.04)$
Savings > €10k	–	$$0.19 \; (0.04)^{***}$$
Real estate > €10k	–	$$0.23 \; (0.04)^{***}$$
VVD supporter	–	$$0.18 \; (0.05)^{***}$$
Wave 2	$$0.10 \; (0.06)^{*}$$	$$0.33 \; (0.05)^{***}$$
Wave 3	$$0.30 \; (0.05)^{***}$$	$$0.52 \; (0.05)^{***}$$
Wave 4	$$0.47 \; (0.06)^{***}$$	$$0.56 \; (0.05)^{***}$$
Intercept	$$1.85 \; (0.06)^{***}$$	$$1.13 \; (0.06)^{***}$$
Adj. $\hbox {R}^2$	0.23	0.12
Observations	5121	4991

Note: $$^{***}p<0.01$$, $$^{**}p<0.05$$, $$^*p<0.1$$. OLS estimations. Standard errors shown in parentheses. Dependent variable for both models: Bitcoin positive impact (0$$-$$6.5 for the UK, 0–7 for the Netherlands). All explanatory variables are binary. All estimations employ survey weights. Wave 1 is the reference category for the survey wave variable. The first model uses UK data, the last Dutch data

We see a quite some similarity with crypto ownership here. The positive Bitcoin narrative is shared more by the young, by men, by homeowners, and by Londoners (Table 2, model 1). In the Netherlands, we observe a similar picture (Table 2, model 2). Among the privileged groups, men are more likely to view Bitcoin positively; the same observation is made for high-social class citizens in the Netherlands. Higher income, by contrast, does not translate into more favorable opinions on Bitcoin.

Table 2 (model 2) suggests that the typical Dutch person who is susceptible to the positive Bitcoin story is a wealthy young man who is high-class, owns his own home, and votes VVD. It is plausible to argue that, of all possible groups, this *jeunesse d’or* is a likely group to effectively spread the Bitcoin evangelism to the rest of the population. This might make the positive side of Bitcoin interesting, for instance, for the pan-European Volt party, and for other parties that target young voters. At the same time, it is unlikely that the growing group that has environmental concerns about Bitcoin will soon be swayed, and millions of elderly probably never will. This could apparently make the negative aspects of Bitcoin appealing to parties with an older and greener support base. It may explain why Dutch Labor has raised the most vocal opposition against Bitcoin. Their median voter age of 63 years in the 2021 General Election was among the highest of 17 parties (Rekker and de Lange 2021). In the long run, however, PvdA (as well as CDA and 50PLUS) will need to cater to young voters, so it might be strategic for them not to raise the Bitcoin issues at this time.

All in all, our analyses reveal two findings in both countries. First, there are clear differences between socio-demographic groups in relation to crypto ownership: in general, individuals from traditionally privileged groups (e.g., men, those with higher income, those from a higher social class, and real estate owners) are more likely to own or have owned crypto (see Table 1). Given that parties generally tend to respond more to the interests of the affluent, this opens up the possibility for unequal representation. Second, there is a socio-demographic divide regarding Bitcoin’s perceived positive impact (see Table 2): not only do privileged groups have distinct evaluations, but they are also more sympathetic toward crypto. This aligns with Weber’s (2020) prediction of no unequal representation when significant differences between social groups exist, and elites hold a more positive view. Although the low salience of the issue and the structure of crypto ownership would suggest that elite interests should dominate, this specific alignment of public opinion might prevent that. Moreover, the age divide we observe in both Tables 1 and 2 could make parties hesitant to address the Bitcoin issues. The next section will assess whether parties respond in line with these findings.

### Do parties use the Bitcoin issue strategically?

**Figure 4:**
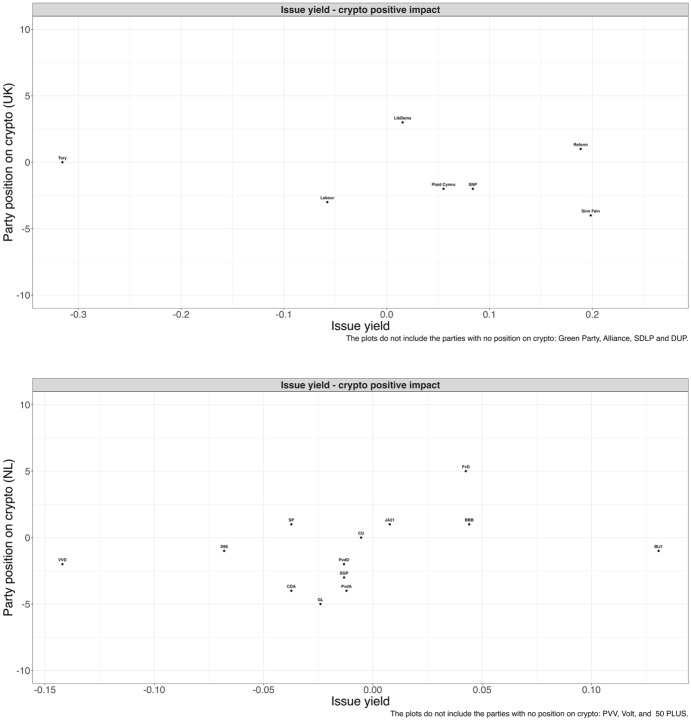
Party position on Bitcoin and issue yield in the UK and the Netherlands

**Figure 5:**
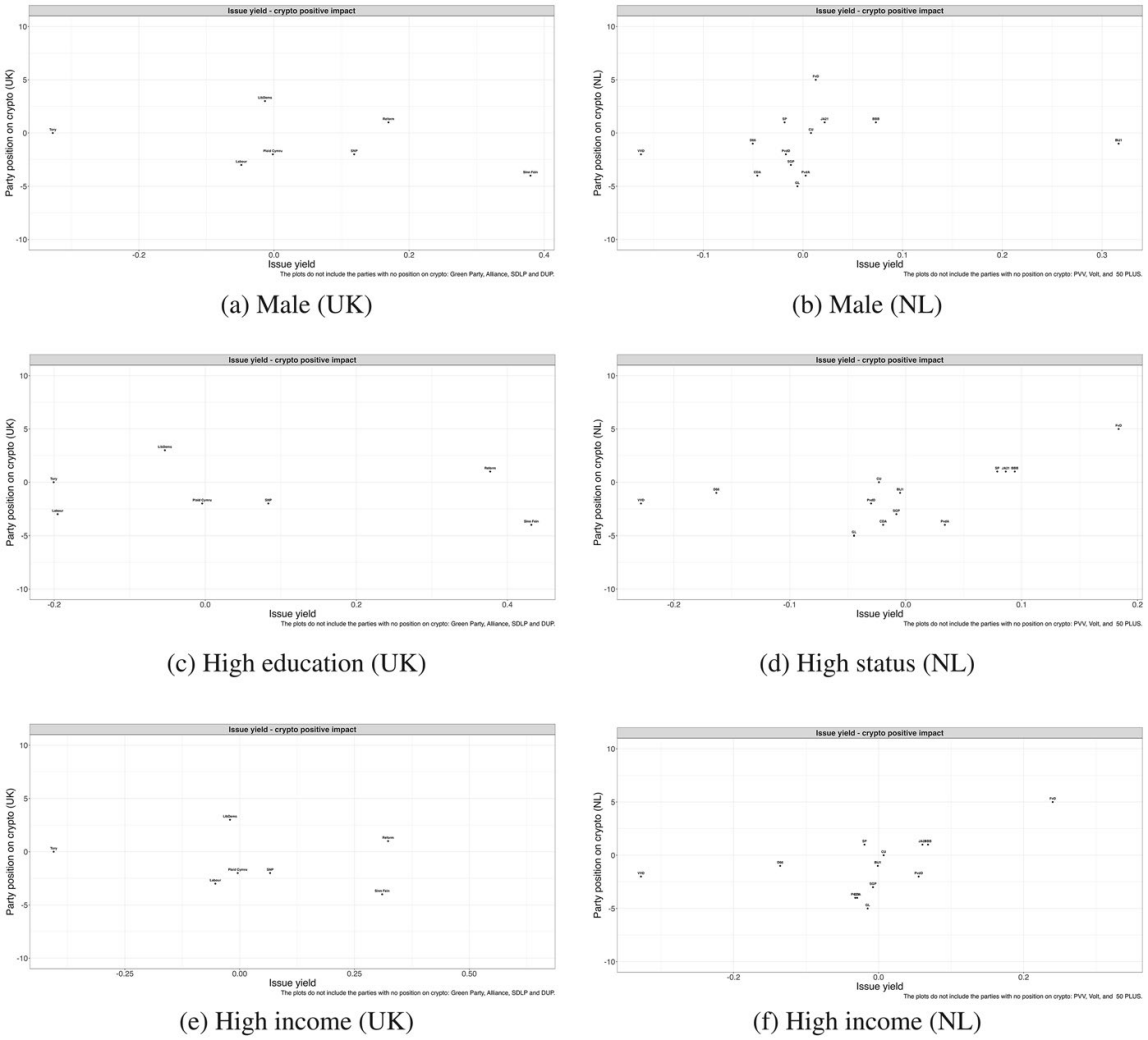
Party position on Bitcoin and issue yield in the UK and the Netherlands (the privileged groups)

Finally, we perform issue yield calculations for each party*index combination.^14^ In Figure 4, we show the results for the whole electorate, whereas in Figure 5, we focus on the privileged groups – the men, educated, and affluent. For simplicity, we present only the figures for the first index (a positive view of Bitcoin), but the full figures are reported in Appendices I and J. Given the novelty of the issue and the low salience of the Bitcoin topic, these results cannot constitute a test of the issue yield theory. Nonetheless, this exercise could provide valuable insights through the analysis of the realized values of issue yield. Specifically, this could tell us whether some parties have a particularly strong incentive to emphasize the issue and, more broadly, whether there is politicization potential around Bitcoin. Small issue yield scores could provide an initial explanation for why parties generally do not promote their stance on Bitcoin.

In Figures 4 and 5 we have plotted, for each party, its issue yield for the positive dimension on the *x* axis against its Bitcoin position on the *y* axis. For parties that do not have a position on Bitcoin we have calculated issue yield scores although they are not displayed in the figure (see Figures I.2 and J.2 in Appendices I and J for figures with parties without position coded “zero”). If a party has high issue yield regarding the positive component, this means that the party has an incentive to take up a position favoring Bitcoin.

We focus first on Figure 4. In the UK case, the issue yields are generally very small, varying between $-$0.06 for Labor and 0.19 for Sinn Fein. The only party for which we see a more sizeable issue yield ($-$0.32) is Tory, but the negative sign suggests that the party should rather be critical of Bitcoin. In the Netherlands, the issue yield scores are equally tiny, varying between $-$0.14 for VVD and 0.13 for BIJ1. These small issue yield values reveal that no party in the UK or Netherlands has a particularly strong incentive to emphasize Bitcoin issues, which could account for why we have seen that attempts at politicization have been limited.^15^ These findings are aligned with the results shown in Table 2 and with Weber’s (2020) theory: as there are clear differences between the privileged and less privileged groups in their positive assessment of Bitcoin, and the better off are more sympathetic to crypto, so parties have no strategic interest to put the issue on the public agenda. Additionally, we do not see evidence that right-wing parties are more supportive of Bitcoin: in fact, both the Tories and VVD have the lowest issue yield, which seems to be partly reflected in the party position only in the Netherlands. At the same time, left-wing parties (e.g., Labor, GL, PvdA) have generally negative issue yields, which is aligned with the parties’ critical stance on Bitcoin. However, these ideological differences are of limited importance: as explained, what stands out in Figure 4 is the reduced strategic opportunity presented by the Bitcoin issue, which is generally reflected in the lack of party attention to the topic.

In Figure 5, we zoom in on the relationship between issue yield and party positions among the groups identified as traditionally privileged by Weber (2020): men, the educated, and the affluent. Again, we focus on the positive Bitcoin impact index. The general picture is not different from the one in Figure 4. Even among privileged groups, the issue yield for the parties in the UK and Netherlands is small.^16^ There seems to be little to electorally gain for parties that focus on Bitcoin’s positive effects, even among sympathetic groups – hence their reluctance to engage with the topic. This specific distribution of public opinion, both among the general public and privileged groups, acts as a brake against unequal representation. In both countries, right-wing parties such as the Tories and VVD have the lowest, although substantively small, issue yield, making them less supportive of the topic, contrary to theoretical expectations. Left-wing parties, such as Labor, GL, or PvdA, generally have negative and/or small issue yield scores. Overall, the small realized issue yield values are more relevant than the ideological component, even among the privileged groups.

## Conclusion

“The political spectrum will rotate. Imagine adding a splash of yellow, so that red versus blue becomes orange versus green (...) Bitcoin Orange versus Dollar Green. On the Dollar Green side are quite a few Republicans, a lot of that security state people, military people, neo-cons, folks who (...) at the end of the day, will choose the American flag and the US government and so on. On the other side is Bitcoin Orange, and lots of Democrats will choose this side.”^17^ This is what US entrepreneur and investor Balaji Srinivasan forecast about politics in his country. To us, this seems an improbable scenario for the near future. Yet, in two other countries, the UK and the Netherlands, we detect some early indications of Bitcoin giving rise to new policy issues among voters. We also see that most parties have reacted, at least, by taking up a Bitcoin position – even if not by advertising that position.

The many-sided nature of Bitcoin allows us to investigate whether and how a new set of policy issues creates opportunities and risks for parties. To the best of our knowledge, we are the first to explore the political consequences of crypto-related issues. Although conducted in only two countries and offering only descriptive evidence, our work tracks the political development of that new set of issues and the relevance of Bitcoin to political representation. We illustrate how parties experiment with new issues. We find that some conditions are met for Bitcoin to gain political traction, but we also find key constraints.

At the demand side of the electoral market, the number of crypto holders substantially increases over time, even during a bear market period. The number of voters who take up a position (either positive or negative) on several issues is also growing. Furthermore, we observe the emergence of socio-demographic and political divisions, especially concerning age and gender. Note that, contrary to what some of the literature suggests, Bitcoin seems neither “inseparable from right-wing philosophy” (Tseng et al. 2022, 91) nor “right-wing extremism” (Golumbia 2016). In fact, support among progressives (who vote for parties such as Greens and BIJ1) is strikingly high. This is partly a composition effect, as many of their supporters are young – and age is the most powerful predictor here. It may also stem from their affinity with decentralized systems or their hopes of curbing the power of banks and financial institutions. However, this support is being undercut by an increase in worries about Bitcoin’s impact on climate change.

At the supply side, many parties abandon caution and develop a position on the issue. Ardent support comes from far right, pro-privacy, and libertarian parties, perhaps related to the notion of separation of money and government, which fits their ideology. Although most parties turn out to have a position when asked, only few of them have signaled salience. One reason for this is that the electoral gains from talking about Bitcoin’s (positive) effects are limited, as observed in the small realized issue yield scores. Even the distinction between economic right and left parties is overshadowed by these low issue yields. Another reason is the high electoral risk, as age cuts across the socio-economic basis of Bitcoin support, leading to internal divisions within most parties and causing them to keep a low profile. As a result, for most political parties, Bitcoin represents a low-gain, high-risk set of issues.

Despite the low salience of the topic, parties lack incentives to cater even to the interests of privileged groups (male, educated, or affluent voters), who are more likely to own or have owned crypto and be sympathetic toward Bitcoin. This is because, as theorized by Weber (2020), bias in political representation will be small if voter views differ markedly between social groups and if support for a goal is higher among a traditionally privileged group than among a traditionally marginalized group. These two conditions find empirical support in our data, which explains why we do not see significant discrimination in party responsiveness between groups. Overall, our work suggests an answer to the puzzle of why the party system has not fully embraced elite interests on this issue. While the low salience may suggest that elite interests should dominate, the specific alignment of public opinion prevents this from happening.

Parties may begin to politicize Bitcoin as the issue gains traction among the public. For instance, as the rate of crypto adoption increases, age-related divisions within the electoral base of parties may diminish, encouraging them to address the issue. Additionally, broader crypto adoption could lessen the differences related to Bitcoin between traditionally privileged and marginalized groups. This might create opportunities for politicization, and also for unequal representation. Moreover, as the constraints discussed above loosen, parties that are at a disadvantage on the dominant axis of political competition may become issue entrepreneurs (Hobolt and de Vries (2015) and view Bitcoin as a viable topic.

Finally, it is also possible that Bitcoin will continue to offer limited electoral opportunities for parties. We must entertain the possibility that Bitcoin will never reach any political significance, as some of the scenarios laid out by Warren (2023) imply. For example, if 32% of our Dutch respondents and 44% of our UK respondents are right and Bitcoin will soon prove worthless, Cruz, Warren, and other politicians will have to find another issue to battle over.

## Supplementary Information

Below is the link to the electronic supplementary material.

Supplementary file 1 (pdf 272 KB)

## Data Availability

The data and replication code will soon be available on the authors' website.

## Footnotes

1 Cruz made these statements during a keynote address at a Heritage Foundation event. Source: Daniel Kuhn, CoinDesk, 24 May 2022; the current Texas Governor is Greg Abbott.

2 Justin Trudeau on X (then still known as Twitter) in response to remarks by Pierre Poilievre, 13 September 2022.

3 Although Bitcoin did not come out of nowhere but was inspired by various precursors (Van Wirdum 2023) it clearly is the first that millions of people worldwide have caught on to.

4 The most prevalent area of interest relates to the impact of media discussions on Bitcoin attention and pricing, see examples in Appendix A.

5 See Appendix E.2 for details.

6 For survey information, see Appendix F.

7 The construction of the four indices is explained in Appendix F.

8 Regarding the demand side, we discuss the positions of 32 parties in these two countries on Bitcoin in detail in Appendix E. We find that while most parties can provide their position, only a few actively advertise it. This provides *prima facie* evidence that political parties in the two countries do not perceive Bitcoin as an electoral opportunity.

9 We actually asked about possession of “cryptocurrencies such as Bitcoin,” but the overwhelming majority of crypto owners (also) holds Bitcoin. It is certainly possible that some crypto owners refuse to reveal their ownership. However, additional analyses suggest that very few, if any, do so. For example, we have asked about four kinds of other possessions as well (savings, real estate, stocks, and gold) in random order, and very few respondents refuse to answer only the crypto question, and there are no order effects. As another example, we benchmark our findings with other (also much larger) surveys from reputable research firms and of all 8 series of observations, one of our surveys comes out highest and the other lowest, see Figure G.2 (Appendix G). We also show the evolution of the crypto ownership for the adult population of the two countries in Figure G.1 (Appendix G).

10 We also find that, in the UK, crypto ownership is moderately correlated with gold possession (r = 0.41). The correlation is much weaker in the Netherlands (r = 0.12). The share of citizens who hold crypto varies from 38% (wave 1) to 57% among those who own gold in the UK, and from 8% to 18% in the Netherlands. This suggests a breakaway by British younger cohorts from common ways of preserving their wealth: instead of savings, real estate, or stocks, they rely on gold and crypto.

11 In Appendix H, we also zero in on those who hold large crypto bags. Moreover, we measure how many of the supporters of each party are what we provisionally refer to as “Bitcoiners” – i.e., citizens who view Bitcoin both as important and as a force for good.

12 See, for instance, a 2023 report by KPMG US on Bitcoin and ESG: https://advisory.kpmg.us/articles/2023/bitcoin-role-esg-imperative.html.

13 In waves 3 and 4 of the survey in each country we ask perceived salience for five sets of issues (environment, economy, immigration, foreign affairs, and financial innovation – see Appendix H for details). What we report here are the proportions of voters who indicate “7” on a scale from 1 (“not important at all”) to 7 (“extremely important”) for “environmental issues (e.g., climate change)” in the most recent wave, wave 4 (June 2023).

14 We have to leave out five small Dutch parties (BVNL, DENK, LP, PP, and NSC), as we have too few respondents who report that they support them to calculate issue yield.

15 We reach similar substantive conclusions by looking at the other three indices – see Figure I.1.

16 This holds for the three other indices as well, as shown in Figure J.1.

17 Quote from Balaji Srinivasan, 19 July 2022. See https://www.reddit.com/r/Bitcoin/comments/vy0yxl/balaji_srinivasan_predicts_the_us_political/.

## References

[CR1] Bohr, Jeremiah, Masooda, Bashir. 2014. “Who uses Bitcoin? An exploration of the Bitcoin community.” In *2014 Twelfth Annual International Conference on Privacy, Security and Trust*, 94-101. IEEE.

[CR2] Breedlove, Robert, J. M., Bush, Gabe Higgins, George Mekhail, Lyle Pratt, Jimmy Song, Julia Tourianski, Derek Waltchack. 2020. *Thank God for Bitcoin: The creation, corruption, and redemption of money*. Whispering Candle.

[CR3] van de Wardt, Marc, Catherine de Vries, and Sara Hobolt. 2014. Exploiting the cracks: Wedge issues in multiparty competition. *The Journal of Politics* 76 (4): 986–999.

[CR4] Elsässer, Lea, Svenja Hense, and Armin Schäfer. 2018. *Government of the People, by the Elite, for the Rich: Unequal Responsiveness in an Unlikely Case*. MPIfG discussion paper.

[CR5] Elsässser, Lea, and Armin Schäfer. 2023. Political inequality in rich democracies. *Annual Review of Political Science* 26 (1): 469–487.

[CR6] Gilens, Martin. 2005. Inequality and democratic responsiveness. *Public Opinion Quarterly* 69 (5): 778–796.

[CR7] Golumbia, David. 2016. *The politics of Bitcoin: Software as right-wing extremism*. Minneapolis: University of Minnesota Press.

[CR8] Hobolt, Sara, and Catherine de Vries. 2015. Issue entrepreneurship and multiparty competition. *Comparative Political Studies* 48 (9): 1159–1185.

[CR9] Homola, Jonathan. 2019. Are parties equally responsive to women and men? *British Journal of Political Science* 49 (3): 957–975.

[CR10] Rekker, Roderik, and de Lange, Sarah. 2021. “De vergrijzing van de PvdA: oorzaken en oplossingen.” *Wiardi Beckman Stichting (wbs.nl)*.

[CR11] Rosset, Jan, and Christian Stecker. 2019. How well are citizens represented by their governments? Issue congruence and inequality in Europe. *European Political Science Review* 11 (2): 145–160.

[CR12] Schakel, Wouter. 2021. Unequal policy responsiveness in the Netherlands. *Socio-Economic Review* 19 (1): 37–57.

[CR13] Schakel, Wouter, and Daphne Van Der Pas. 2021. Degrees of influence: Educational inequality in policy representation. *European Journal of Political Research* 60 (2): 418–437.

[CR14] De Sio, Lorenzo, and Till Weber. 2014. Issue yield: A model of party strategy in multidimensional space. *American Political Science Review* 108 (4): 870–885.

[CR15] De Sio, Lorenzo, and Till Weber. 2020. Issue yield, campaign communication, and electoral performance: A six-country comparative analysis. *West European Politics* 43 (3): 720–745.

[CR16] De Sio, Lorenzo, Mark Franklin, and Till Weber. 2016. The risks and opportunities of Europe: How issue yield explains (non-)reactions to the financial crisis. *Electoral Studies* 44: 483–491.

[CR17] Sylvester, Christine, Zachary Greene, and Benedikt Ebing. 2022. "ParlEE plenary speeches data set: Annotated full-text of 21.6 million sentence-level plenary speeches of eight EU states."

[CR18] Tseng, Darren, Stephen Diehl, and Jan Akalin. 2022. *Popping the Crypto Bubble: Market Manias, Phony Populism*. Techno-Solutionism: Consilience Publishing.

[CR19] Van Wirdum, Aaron. 2023. *The Genesis Book: The Story of the People and Projects that Inspired Bitcoin*. Bitcoin Magazine.

[CR20] Warren, Micah. 2023. *Bitcoin: A Game-Theoretic Analysis*. Walter de Gruyter.

[CR21] Weber, Till. 2020. Discreet inequality: How party agendas embrace privileged interests. *Comparative Political Studies* 53 (10–11): 1767–1797.

