# Supplementary material for: Privileged interests on the party agenda: Bitcoin-related issues in two countries since 2020
Source: Acta Polit. 2024 Dec 23;61(2):393–413. doi: 10.1057/s41269-024-00380-4 (PMC12999464; doi:10.1057/s41269-024-00380-4)
Supplement: Supplementary file 1 — (pdf 272 KB) [file 41269_2024_380_MOESM1_ESM.pdf]

Online Appendix
Privileged interests on the party agenda: Bitcoin-related issues in two countries since 2020

Appendices - Table of contents

A Studies on the impact of media discussions on Bitcoin attention and pricing 2

B The 32 parties under study 3

C Party answers to our question on their position on Bitcoin 4
C.1 United Kingdom 4
C.2 Netherlands 5

D Bitcoin mentioned in Parliament 10

E The position of political parties on Bitcoin (The demand side) 12
E.1 Party positions on Bitcoin 18
E.2 Inter-coder reliability 19

F Details on the surveys and index construction 20

G Crypto ownership in the UK and the Netherlands since 2017 22

H Analysis of large crypto owners and Bitcoiners 25

I Party position on Bitcoin and issue yield (full samples) 27

J Party position on Bitcoin and issue yield (the privileged groups) 29

A Studies on the impact of media discussions on Bitcoin attention and pricing

The most prevalent area of interest relates to the impact of media discussions on Bitcoin attention and pricing (Mai et al. 2018; Matta et al. 2015; Mai et al. 2015; Burnie and Yilmaz 2019; Philippas et al. 2019; Béjaoui et al. 2021; Poongodi et al. 2021; Urquhart 2018). Among these, Mai et al. (2018) use textual analysis and vector error correction models to explore the dynamic interactions that link social media and the monetary value of Bitcoin. Here, social media sentiment is found to be an evident predictor in determining Bitcoin's value, where the effects are primarily driven by the silent majority of users whose contributions amount to less than 40 percent of total messages. Urquhart (2018), on the other hand, focuses on the drivers of media attention to Bitcoin, finding evidence through Google Trends that previous-day volatility and search volume are significant drivers of attention to Bitcoin.

B The 32 parties under study

Table B.1: The 32 parties under study

Country	Abbreviation	Party name	Vote share
United Kingdom	Tory	Conservative Party	44
United Kingdom	Labour	Labour Party	32
United Kingdom	LibDem	Liberal Democrats	12
United Kingdom	SNP	Scottish National Party	4
United Kingdom	Green	Green Party of England and Wales	3
United Kingdom	Reform	Reform UK	2
United Kingdom	DUP	Democratic Unionist Party	1
United Kingdom	SF	Sinn Féin	1
United Kingdom	PC	Plaid Cymru	0
United Kingdom	SDLP	Social Democratic and Labour Party	0
United Kingdom	Alliance	Alliance Party of Northern Ireland	0
Netherlands	VVD	People's Party for Freedom and Democracy	22
Netherlands	D66	Democrats 1966	15
Netherlands	PVV	Freedom Party	11
Netherlands	CDA	Christian Democratic Appeal	10
Netherlands	SP	Socialist Party	6
Netherlands	PvdA	Labour Party	6
Netherlands	GL	Green Left	5
Netherlands	FvD	Forum for Democracy	5
Netherlands	PvdD	Animal Rights Party	4
Netherlands	CU	Christian Union	3
Netherlands	Volt	Volt Netherlands	2
Netherlands	JA21	The Right Answer 2021	2
Netherlands	SGP	Reformed Political Party	2
Netherlands	DENK	Think [Dutch meaning] / Equal [Turkish]	2
Netherlands	50Plus	50Plus	1
Netherlands	BBB	Farmer-Citizen Movement	1
Netherlands	BIJ1	Together	1
Netherlands	PP	Pirate Party	0
Netherlands	LP	Libertarian Party	0
Netherlands	BVNL	The Netherlands, Ltd.	*est.d later*
Netherlands	NSC	New Social Contract	*est.d later*

Note: Reported here is the vote share (in percentages nationwide) in the most recent first-order election at the time of conducting the study (Summer 2023): the 2019 election to the House of Commons in the UK, and the 2021 election to Second Chamber of the Dutch National Parliament, respectively.

C Party answers to our question on their position on Bitcoin

Request sent to 29 parties on 6 June 2023

Request sent to 1 party on 13 June 2023 (=request to PVV)

Reminder sent to 20 parties on 13 June 2023

Second reminder sent to 13 parties on 20 June 2023

Second reminder sent to 1 party on 22 June 2023 (=reminder to 50Plus)

Request sent to 1 party on 24 August 2023 (=request to NSC)

Request sent to 1 party on 28 August 2023 (=request to LP)

Reminder sent to 2 parties on 20 September 2023 (=reminder to NSC and LP)

C.1 United Kingdom

Alliance: (Helen McCann, Support Services Officer, via email on 6 June 2023) “I have checked with our Policy Team and the Alliance Party does not have a position on Bitcoin.”

Reform UK: (National Support Team, via email on 7 June 2023) “As a political party based on Classical Liberal principles, we are reluctant to take a hard position on Bitcoin. We believe that those trading in Bitcoin, Ethereum, other crypto currencies and Altcoins should be equipped to make responsible decisions and informed choices. As long as this is the case, we support the right of individuals to become market participants, as they see fit.”

Labour: (Lord Iain McNicol of West Kilbride, Crypto and Digital Assets APPG Treasurer, via email on 20 June 2023) “This is a bit wider than specifically Bitcoin, but hope it is of some use”.

- “Labour would be serious about attracting FinTech companies to the UK, by building a regulatory regime that supports innovation to safely harness blockchain technologies and our ambition to make Britain the homegrown start-up hub of the world”.

- “Millions of British consumers’ savings have been put at risk by the collapse of cryptocurrencies while crypto related scams have hit record levels. Despite this, the Conservatives continue to promote cryptocurrency gimmicks”.
- “The Government proposed, then backtracked on poorly judged plans for the Royal Mint to produce a non-fungible token”.
- “Labour has been calling for tougher regulation for months, yet all the Conservatives are promising is further consultations – we need action now”.

Green Party: (Cath Miller, Chief of Staff of MP Caroline Lucas, via email on 27 June 2023) “I am not aware that the Green Party has a formal policy on Bitcoin”.

C.2 Netherlands

GroenLinks: (Valentina, via the official party Whatsapp account on 6 June 2023) “The economic added value of Bitcoins and cryptocurrencies more generally, is very limited. Also, it does little for innovation. GroenLinks thinks trading cryptocurrencies should be labelled ‘gambling’. We also think that the heavy environmental impact associated with mining should be restricted. A ban is difficult in this case; it can be made less attractive. For instance, by means of a heavy taxation, which can, de facto, exert a similar kind of effect as a ban. Ideally, we would make Europe-wide agreements”.

PvdA: (Sytze, via the official party Whatsapp account on 6 June 2023) refers to the text “Drie redenen waarom de overheid moet ingrijpen bij de Bitcoin”, which translates as “Three reasons why the government should intervene in Bitcoin”.

JA21: (Herben Baan, policy officer, via email on 7 June 2023) “JA21 is a proponent of innovations within the financial domain such as blockchain. This said, there must be adequate regulation in this domain, which means that central banks should be able to have a monetary policy. If in

addition to that cryptocurrencies were to develop into legal tender, that would surely be a valuable addition to give also consumers freedom with regard to payment options".

ChristenUnie: (Job Schutte, national parliamentary group officer, via email on 8 June 2023) "I have checked with my Finance colleague, and he says that, in principle, we are not negative towards Bitcoin, and it seems a fine means of payment. This said, we have some concerns about the heavy energy use and the fact that the crypto market is not regulated, which leads to cowboy behaviour at their market and gamification through which youth invest more than is responsible and so on".

SP: (Dilan, via email on 9 June 2023) refers to the text "Breek de bank met Bitcoin?" which means "Breaking the bank with Bitcoin?"

Volt: (Rijk, general mail manager, via email on 9 June 2023) "Volt does not yet have a specific position on Bitcoin or on any other cryptocurrency".

PvdD: (Marie de Vries, public communications officer, via email on 12 June 2023) "Bitcoin (and Bitcoin-like payment systems) itself has become a speculative medium, fuelling bubbles. As such, Bitcoins pose one of many risks to the stability of the already unstable financial system. In addition, Bitcoins slurp energy. It is therefore a polluting means of payment. However, not all is doom and gloom. Behind the Bitcoins is a technology that, like any technology, can be used for good and for evil: blockchain. Blockchain allows transactions to be recorded immutably and completely in successive steps, without being easily traceable to people's identities. This offers possibilities for reliably recording the origin of products or recording CO2 emissions. Blockchain can therefore provide more transparency in production chains. And this in turn could contribute to corporate social responsibility and further sustainability of the financial sector".

D66: (Noa, via the official party Whatsapp account on 13 June 2023) "The current position of D66 is the following. We see the value of technical innovations in the financial sector, but also see the risks that Bitcoin brings around the finances of vulnerable groups. That is why it is good that the MiCA Regulation is now taking a first step towards better enforcement, but during the debate on this we also asked questions about whether this is strict enough. In addition, we see that the stability of cryptocurrencies still leaves much to be desired, so it remains important to look at the opportunities while mitigating the risks as much as possible. We don't want to stop people from experimenting with cryptocurrencies, but we want to do it in a safe way".

PVV: (public communications of the national parliamentary group, via email on 14 June 2023) no clear answer, for all policy positions it is referred to the general website, the manifesto, and parliamentary debates, which mention Bitcoin only once as far as we can tell, a reference to money laundering using Bitcoins and using Bitcoin ATMs.

BVNL: (Stef Kleine Staarman, parliamentary group officer, via email on 14 June 2023) "We are of the opinion that everyone should be free to choose whether to invest in Bitcoin. We are therefore not in favour of a ban on Bitcoin".

FvD: (Floris, Team Webcare, via email on 14 June 2023) "FvD is unabatedly positive towards Bitcoin".

BIJ1: (Kiara Grouwstra, party office, via emails on 14, 22, and 29 June 2023) "With a view of climate goals not a fan of crypto currencies (especially not in the traditional proof-of-work category) such as Bitcoin".

BBB: (party office, via email on 19 June 2023) "BBB allows people ('lets people free') to trade in crypto coins but we are sceptical about a digital euro".

PP: (Matthijs Pontier, chairman of the party's parliamentary group in the Amstel, Gooi en Vecht Water Board, via Twitter Messenger on 20 June 2023) refers to statement in the 2021 Bitcoin.nl survey, position has not changed.

SGP: (Sander Bossenbroek, policy officer, on behalf of MP Kees van der Staaij, via email on 21 June 2023) "The SGP sees opportunities in Bitcoin, but also dangers. It can offer opportunities for innovation or the development of our financial system, for example. But there are also many dangers involved. For example, for consumers, who often purchase such crypto coins without much knowledge. The fact that there is still little regulation is also worrying. In addition, the question is what exactly Bitcoin adds to our monetary system. Finally, you also see that it is used in criminal circles, which of course also worries us. In short, the SGP is critical of cryptocurrencies such as Bitcoin".

DENK: (Piotr van Rij, senior policy officer, via email on 22 June 2023) "there is no specific position on Bitcoin in the DENK's current election program".

CDA: (MP Evert Jan Slootweg via telephone on 3 July 2023) "five points:"

- "In 2014-2015 we saw that many people invested money in it. At the time it only went up, and we wondered how to tax their surplus".
- "What is the underlying value of this? It is not physical, there is a lot of price manipulation, it travels across borders, so our starting point was that we had to approach this EU-wide and soon we will have the MiCA Regulation".
- "How do we protect adolescents? It is an intangible asset. So many youngsters are invested in it, although they should not do so as long as they do not understand it. There is FOMO [fear of missing out]. We should do something about their being lured into this. We should consider

banning advertisements, especially online because it is tailored to social media users".

- "We have to consider its ecological footprint. A lot of energy is being used for this. How effective is that? Bitcoin is perhaps the most energy slurping of all coins. This does not square with greening and the energy transition".

- "We must consider the effect on the real economy. Financial stability is important, we have to protect our system. When banks can offer Bitcoin services to customers, we are afraid of a legitimation effect. This brings along additional demand and there will be quite a bit of volatility".

[additional points when asked: "A Bitcoin ban is not possible, and I am not sure if I would want a ban, but I do want stricter regulation. If there are any opportunities, then they will manifest themselves in a better way if there is regulation. We should regulate speculative aspects so as to build trust and reliability. As a representative, I cater to general interests, not special interests of a group of speculators. Bitcoin is not very different from other coins. The notion of a separation of state and money does not appeal to us. As soon as one issues a coin one has a responsibility toward society, and it should be regulated".]

D Bitcoin mentioned in Parliament

Using the ParlEE dataset (Sylvester et al. 2022) we found that before 1 January 2020, Bitcoin was mentioned 13 times in the British and 104 times in the Dutch National Parliament.

Table D.1: Bitcoin mentioned in the British National Parliament before 1 January 2020

**Date**	**Speaker**	**Party**	**Sentence**
17/4/2013	Chris Leslie	Lab	I am sure that whether it be in Bitcoins, gold or shares, bankers will be ingenious in how they pay and reward themselves.
20/11/2014	Steven Baker	Con	Positive Money proposes the complete nationalization of the production of money, some want variations on a return to gold, perhaps with free banking, and some want a spontaneous emergence of alternative moneys like Bitcoin.
20/11/2014	Steven Baker	Con	What we see today is that, with alternative currencies such as Bitcoin spontaneously emerging, it is now possible through technology that, within a generation, we will not all be putting our money in a few big mega-banks, held as liabilities, issued out of nothing.
20/11/2014	Steven Baker	Con	Imperfect and possibly doomed as it may be, Bitcoin shows us that peer-to-peer, non-state money is practical and effective.
05/1/2016	Gisela Stuart	Lab	We also read about the emergence of the Bitcoin market.
11/7/2016	Eilidh Whiteford	SNP	With many economists predicting a further recession as a consequence of Brexit, and the pound now less stable than Bitcoin, will the Secretary of State assure me that he will not allow those on low and middle incomes to bear the brunt of further economic downturn?
13/9/2016	Fiona Mactaggart	Lab	But what about the sites that do not depend on any UK-based credit card companies, or that use Bitcoin or other non-UK-based financing systems?
28/11/2016	Claire Perry	Con	Secondly, we know that many sites are not reliant purely on financial transactions coming through the sorts of sites discussed in the Bill, given that there are systems such as Bitcoin and other forms of revenue generation.
22/11/2017	Luke Graham	Con	Digital pervades every aspect of our lives today: in communication, through email, Facebook and Twitter; in banking, business and bitcoin; in farming, as farmers fulfill their CAP obligations; in retail, as retailers try to reach domestic and international customers; and in benefits, welfare and healthcare, all of which are moving online.
21/2/2018	Rachel Maclean	Con	I hope that, when he winds up the debate, the Minister will touch on the important issues of cryptocurrencies and bitcoin which, I believe, are not currently covered by regulation.
03/5/2018	Jessica Morden	Lab	My constituent Malcolm Richards was the victim of a financial scam advertised on the internet, losing £39,000 in a bitcoin scam that purported to be backed by members of Dragons' Den.
22/5/2018	Ben Wallace	Con	People can sit at home and order and deal drugs, and they can launder the money almost instantaneously through Bitcoin and elsewhere.
20/11/2018	Eddie Hughes	Con	I am delighted to say that on Thursday I am going to have lunch with Dr. Craig Wright, one of the people associated with the creation of bitcoin, which celebrated its 10th birthday recently.

Table D.2: Bitcoin mentioned in the Dutch National Parliament before 1 January 2020

**Party**	**Dates**	**Sentences**	**Speakers**
PVV	22/11/2016	1	Dion Graus (1)
VVD	13/12/2016	1	Ockje Tellegen (1), Roald van der Linde (15), Dilan Yesilgöz-Zegerius (1)
	12/12/2017	1	
	31/1/2018	1	
	16/5/2018	10	
	20/11/2019	1	
	03/12/2019	3	
CDA	29/11/2017	2	Madeleine van Toorenburg (3), Ferd Grapperhaus (4), Erik Ronnes (11), Wopke Hoekstra (7)
	30/11/2017	5	
	12/12/2017	2	
	31/1/2018	6	
	21/2/2018	1	
	16/5/2018	9	
PvdA	12/12/2017	9	Henk Nijboer (17)
	31/1/2018	4	
	21/2/2018	1	
	16/5/2018	3	
D66	12/12/2017	7	Wouter Koolmees (5), Jan Paternotte (9), Kees Verhoeven (2), Monica den Boer (1)
	16/5/2018	7	
	25/6/2019	2	
	29/10/2019	1	
SP	12/12/2017	5	Renske Leijten (5), Mahir Alkaya (11)
	16/5/2018	5	
	06/6/2018	1	
	03/12/2019	5	
GL	31/1/2018	3	Bart Snels (3)
DENK	16/5/2018	7	Farid Azarkan (7)
50PLUS	19/9/2019	1	Henk Krol (1)

E The position of political parties on Bitcoin (The demand side)

For the positions of 32 parties in these two countries, see Figure E.1 and Appendix E.1 (Table E.1). From this figure, we can tell that a lot is unclear and ambiguous. This is perhaps unsurprising, given that we focus on a nascent topic. Moreover, we distinguish between salience and position (Table E.1): Whereas most parties can tell us their position, only few of them actually advertise that position.

Just seven parties have taken the initiative to discuss crypto, as far as we can tell: one British party and six Dutch ones. In the UK, only the Conservative Party has done so. In 2022, when he was Chancellor, former Prime Minister Rishi Sunak said that Britain was "open for crypto businesses" and should become a "global hub."[1] The Conservative government claimed it wanted to help companies invest, innovate and scale up. This involves making tax code work more easily for crypto, facilitating cross-border payments, legislation to establish a sandbox, and meetings with business eight times a year along with a two-day "CryptoSprint" conference in May 2022.[2] The Conservatives focused on stablecoins and explicitly stated that Bitcoin will not be legal tender.[3]

Other British parties have just one or two representatives who have spoken out before 2024. On the positive side, we have Reform UK and the Scottish National Party. In 2020, when Reform UK was called Brexit Party, its leader Nigel Farage said that it is "crucially important" for people to

[1]He did so a few months after Christopher Harborne, a prominent crypto investor, had donated £500,000 to the Tories. Source: Alex Daniel, Financial News London (fnlondon.com), 10 August 2022.

[2]Source: "UK aims to become global crypto hub, Exchequer says," reportage by Camomile Shumba at CoinDesk, 4 April 2022.

[3]This said, the Tories are quite divided on Bitcoin. Of the 54 Tory MPs who responded to the survey we commissioned, 29% said the party did not have a position, 20% said they did not know, 13% said 0, 20% said the party mainly saw restrictions (-5 thru -1), and 17% said it mainly saw opportunities (+1 thru +5).

Figure E.1: Party positions on Bitcoin

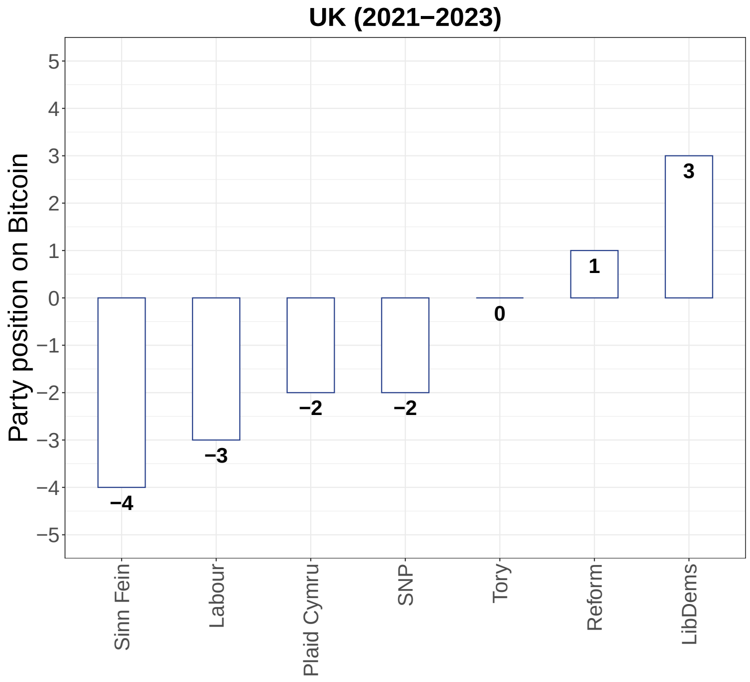

The plots do not include the parties with no position on crypto:
Green Party, Alliance, SDLP and DUP.

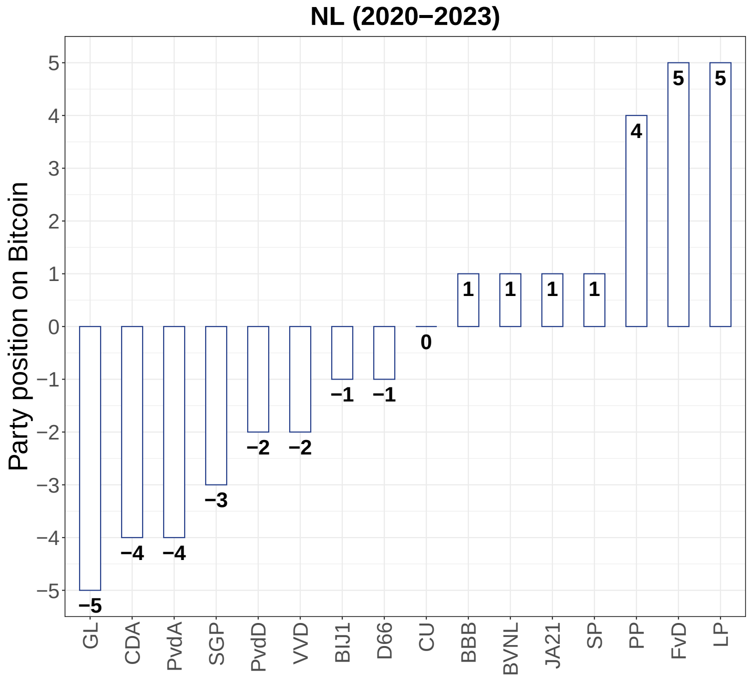

The plots do not include the parties with no position on crypto:
PVV, Volt, 50 PLUS, Denk, and NSC.

For details, see Table E.1 (Appendix E.1).

get into crypto and called Bitcoin the "ultimate anti-lockdown investment."[4] After stepping down in March 2021, Farage headlined the 2022 Bitcoin Amsterdam conference, labelling Bitcoin "the ultimate freedom." The position of the party more broadly is unknown.[5] Turning to the SNP, its MP Lisa Cameron was, in the previous Parliament, the chair of the Crypto and Digital Assets All Party Parliamentary Group. She said in 2022 that she was "not an evangelist… there are opportunities in terms of innovation and job creation. You can't keep your head in the sand and hope it goes away."[6] She wants to ensure that the UK becomes a "global hub for crypto-asset technology."[7]

Again, other UK party members have made sceptical comments. Labour[8] and SF should be mentioned here. In 2018, Labour shadow home secretary Diane Abbott called Bitcoin a "Ponzi Scheme" and said that the party would pass "proper" regulation when in government. Abbott also said she was worried about Bitcoin being used to "fund terrorist activity."[9] "Many are rightly questioning whether crypto has a future at all," Labour Treasury spokeswoman Abena Oppong-

[4]Source: "Nigel Farage pivots to crypto: It was only a matter of time," column by Jamima Kelly in the Financial Times, 3 December 2020.

[5]This said, the Reform party's National Support Team was quick to react to our survey, see Appendix C.

[6]Source: "UK sends mixed messages on becoming a crypto hub," reporting by Josh Adams for BeInCrypto, 24 December 2022.

[7]Source: "British MP Lisa Cameron calls for cryptocurrency regulation," reporting by Sarah Jansen for Crypto News, 2 May 2023.

[8]Of the 38 Labour MPs who responded to the survey we commissioned, 18% said the party did not have a position, 36% said they did not know, 5% said 0, another 37% said the party mainly saw restrictions (-5 thru -1), and 3% said it mainly saw opportunities (+1).

[9]Source: "UK, blockchain and the general election: what you need to know," reporting by Melanie Kramer for Decrypt, 11 December 2019.

Asare said during a debate in Parliament in September 2022.[10] Similarly, a prominent SF politician expressed his concerns. Speaking in a debate in Strasbourg in 2022, MEP Chris MacManus said: "At their worst crypto assets can be pyramid schemes, they are used by criminal gangs for money laundering and to defraud working people and they can waste huge amounts of energy for no purpose. In short, I see little or no social or economic benefit to these tools of speculation (...) short of banning them, they must be regulated."[11]

In the Netherlands, we have found statements that can be related to 13 out of 21 parties yet only six have clearly spoken out.[12] On the critical side, MEP Paul Tang (PvdA) has published several hit pieces against crypto, comparing it to the 17th century Dutch tulip mania. "But then again, tulips are at least pretty." Just as his SF colleague in 2022, Tang welcomed EU regulation in 2023. "Yet unhosted wallets still lurk in the dark and can escape the eye of the law."[13] Similarly, an op-ed written by PvdA MP Henk Nijboer in 2018 demands government intervention in Bitcoin.[14] His SP colleague Mahir Alkaya has also written op-eds. In addition, just as Farage, he featured at the 2022 Bitcoin Amsterdam conference, articulating his nuanced positive stance towards Bitcoin.

[10]Source: "UK sends mixed messages on becoming a crypto hub," reporting by Josh Adams for BeInCrypto, 24 December 2022.

[11]Source: "MacManus welcomes crypto regulation," reporting by Chris MacManus on 1 July 2022 for SinnFein.ie.

[12]Two parties, such as CU and PVV, have a text on their website that mentions Bitcoin but only in passing and in the context of debates about money laundering. Again others, PvdA, CDA, D66, GL, and DENK, have participated in parliamentary debates. However, this does not mean that they signal salience.

[13]Source: "Why the EU is ahead of other jurisdictions on crypto as MiCA set to kick in," Joanna Wright and Eric Johansson reporting for DL News, 19 April 2023.

[14]https://www.pvda.nl/nieuws/drie-redenen-overheid-moet-ingrijpen-bitcoin/, also published as an op-ed in Algemeen Dagblad on 24 January 2018.

In 2022 he also published a book which touches on Bitcoin.[15] At the right side of the political spectrum, JA21 mentioned Bitcoin in its 2021 party manifesto: "Bitcoin and other digital currencies increase freedom of choice. We must therefore embrace innovation from this sector and not regulate it away." Even more pro-Bitcoin, prominent PP spokesperson Matthijs Pontier has favourably discussed crypto on several public occasions, and FvD leader Thierry Baudet publicly applauded a statue for Satoshi Nakamoto in Hungary in 2023[16] and suggested that the government could build coal plants to mine Bitcoin.[17] The libertarian LP stated in 2021 that it argues for research into Bitcoin as a money standard in the Netherlands and making crypto tax free while the party is against identification requirements for unhosted wallets and the registration obligation for starting crypto companies.[18]

Notwithstanding the low salience they attach to it, 23 out of 32 parties have a stance on Bitcoin. The nine that do not hold any position are niche parties that tend to focus, although not exclusively, on a particular issue that is nowhere close to Bitcoin. Of the 23 parties that actually have a position, 13 are negative while 10 are more positive. Just as in the US, the negativity mainly comes from the left side of the aisle. In both countries, perhaps the strongest opposition comes from Labour.[19] This said, all leftist parties are negative except for the far left SP. This is because Mahir Alkaya, the

---

[15] The book, published by Bot Uitgevers in 2022, is entitled "Van wie wordt ons geld?", which translates as "To whom will our money belong?".

[16] See `www.youtube.com/watch?v=_9pMC8Sm8Kw`.

[17] On 6 March 2023, David da Silva Rosa posted a video clip on X in which Baudet suggested this.

[18] See `https://bitcoin.nl/artikel/stemwijzer-verkiezingen-2021-wat-vinden-politieke-partijen-van-bitcoin`.

[19] Labour's Dutch equivalent is the PvdA, and the latter's sister party is GL. They are the most ardent opposition to Bitcoin, together with SF in Northern Ireland and the center-right CDA (which, in a joint initiative with the PvdA, suggested to ban citizens from using their credit card to purchase Bitcoin, see Parliamentary Debates TK 81 11 pp. 2-17, 16 May 2018).

parliament's fintech expert, is cautiously positive and happens to be MP for that party. Christian parties (CDA, CU, SGP) are sometimes neutral and at other times issue warnings against Bitcoin – just as the largest Dutch party, the conservative VVD, does. The British center-right, the Tory party, has a more neutral stance. To the right of these mainstream right parties sits a cluster of staunchly conservative parties (Reform, JA21, BVNL, and BBB) that are all mildly positive. Clearer positive positions range from the nuanced LibDems[20] via the outspoken PP and the "unabatedly positive" far right FvD to the concrete and well-articulated support by the libertarian LP.

[20]The Vice Chairman of the Liberal Democrat Business Network is Mihir Magudia. He is the author of the book "Money Unbound: Bitcoin and the Breaking of the Old World" (2021), which according to its Amazon.com description, tells seven stories about citizens around the world who can benefit from, and succeed with, "Bitcoin and blockchain."

E.1 Party positions on Bitcoin

Table E.1: Party positions on Bitcoin

Party	Our survey 2023	MP survey Parliamentary debates	Bitcoin.nl survey 2021	Bitcoindebuut.nl survey 2020	Overall dummy	Overall average	Overall latest
			**United Kingdom**				
**Alliance**	X				X	X	X
**Reform***	+1				+	+1	+1
**Labour***	-1	-3 (N=38)			-	-2	-3
**Green**	X				X	X	X
**Tory****		0 (N=54)			+	0	0
**LibDem**		+3 (N=2)			+	+3	+3
**SNP***		-2 (N=7)			-	-2	-2
**PC**		-2 (N=2)			-	-2	-2
**DUP**		X (N=3)			X	X	X
**SDLP**		X (N=1)			X	X	X
**SF***		-4 (N=1)			-	-4	-4
			**Netherlands**				
**GL***	-5	-2 (16/5/2018)	-1	-2	-	-3	-5
**PvdA****	-4	-4 (12/12/2017)<br>-4 (16/5/2018)<br>-1 (24/6/2020)<br>-4 (10/5/2023)			-	-3	-4
**JA21****	+1			+3	+	+2	+1
**CU***	0	-3 (20/4/2020)			-	-1	0
**SP****	+1	-2 (12/12/2017)<br>+1 (16/5/2018)<br>+1 (15/7/2019)<br>0 (24/6/2020)<br>+1 (10/5/2023)	+3	0	+	+1	+1
**Volt**	X				X	X	X
**PvdD**	-2		-2		-	-2	-2
**D66***	-1	-3 (12/12/2017)<br>0 (16/5/2018)<br>-4 (10/5/2023)			-	-2	-1
**PVV***	X	+1 (16/5/2018)<br>-2 (10/12/2020)			X	X	X
**BNVL**	+1				+	+1	+1
**FvD****	+5	+3 (17/9/2021)	+3	+3	+	+4	+5
**CDA***	-4	-1 (12/12/2017)<br>-2 (16/5/2018)<br>-4 (10/5/2023)	0	-3	-	-2	-4
**VVD***		-1 (12/12/2017)<br>0 (16/5/2018)<br>0 (15/7/2019)<br>-2 (10/5/2023)	-3	-1	-	-1	-2
**SGP**	-3		0		-	-2	-3
**PP****	+4		+4		+	+4	+4
**DENK***	X	0 (12/12/2017)<br>-1 (16/5/2018)			X	X	X
**BIJ1**	-1				-	-1	-1
**BBB**	+1				+	+1	+1
**50Plus**					X	X	X
**LP****			+5		+	+5	+5
**NSC**					X	X	X

Note: Positions on Bitcoin range from -5 (fully avoid all risks) to +5 (fully seize all opportunities). An “X” indicates that the party does not have a position. Two asterisks mean that a representative of the party has signalled salience. One asterisk that we have found at least one public statement that mentions Bitcoin. Data source Bitcoin.nl: `https://bitcoin.nl/artikel/stemwijzer-verkiezingen-2021-wat-vinden-politieke-partijen-van-bitcoin`. Data source Bitcoindebuut.nl: `https://bitcoindebuut.nl/bitcoin-stemwijzer-verkiezingen-2021-standpunten-politieke-partijen/`.

E.2 Inter-coder reliability

A second coder also coded the responses from the 21 parties that responded to our request. Intercoder reliability was assessed using Krippendorff's α, of which values above .80 and .67 are generally interpreted as respectively good and acceptable reliability (Krippendorff 2018, 241). For variables measured on an ordinal scale, Krippendorff's α also has the advantage that it can take this ordering into account (at the nominal level, values either match or they do not – but with ordered scores, two reported scores of the same unit that do not match may, for instance, both be higher than the reported scores for another unit and coders then may not report exactly the same value, they do reliably recognize that this unit scores higher than another unit; By assessing Krippendorff's α at the ordinal level, this is taken into account). In order to make use of this advantage, we estimated reliability in two steps. First, the original variable was recoded into whether a coder believed the party does or does not have a position on Bitcoin. Both coders perfectly agreed on the same 5 parties that did not have a position on Bitcoin ($\alpha = 1.00$). In the second step, we assessed the reliability of the coded positions of the remaining 16 parties at the ordinal level. With $\alpha = .95$ (95% CI [.88, .99]), this also showed good reliability.

F Details on the surveys and index construction

Data collection in the United Kingdom occurred over four waves: Wave 1 (5-24 February 2021, N=1,642), Wave 2 (3-4 November 2021, N=1,032), Wave 3 (26-30 August 2022, N=1,434), and Wave 4 (9-14 June 2023, N=1,013). The surveys were conducted by Survation, with respondents sampled to reflect nationally representative targets for sex, age group, NUTS 1 region, highest level of qualification, and annual equalized household income. Survey weights were applied in the analysis to ensure representativeness.

Data collection in the Netherlands took place over four waves: Wave 1 (17-21 December 2020, N=1,238), Wave 2 (26 October-5 November 2021, N=1,282), Wave 3 (31 August-14 September 2022, N=1,186), and Wave 4 (5-12 June 2023, N=1,297). The surveys were conducted by the research firm Kantar (now Verian). The samples were representative of the Dutch adult population based on sex, age, education, region, and voting behavior in the most recent Dutch national parliamentary elections at the time (2017 and 2021, respectively). Survey weights were applied to ensure representativeness.

We build our four indices in the following way.

The following 6 statements are part of *the crypto positive impact index*: 1) In 2030, most people will use Bitcoin; 2) Bitcoin helps people in the third world; 3) Some time, Bitcoin will replace the pound/Euro; 4) Savings in Bitcoin are protected from inflation; 5) Bitcoin curbs bank and government power; and 6) In 5 years, 1 Bitcoin will cost over £(or €) 100,000. Answering options to all questions range from 1 (fully disagree) to 7 (fully agree) plus a “don’t know” option.

The following 7 statements and items are part of *the crypto negative impact index*: 1) Bitcoin is mainly for crime and terrorism; 2) Hacks and fraud make Bitcoin vulnerable; 3) Bitcoin is an environmental disaster; 4) Bitcoin worsens economic inequality; 5) Bitcoin price manipulation

is rampant; 6) Soon Bitcoin will prove worthless; and 7) The price of Bitcoin goes up and down sharply. Answering options to all questions range from 1 (fully disagree) to 7 (fully agree) plus a "don't know" option.

The following 4 statements and items are part of *the index on governmental punitive action on crypto*: 1) Bitcoin should be banned by the government; 2) The government should make paying taxes in Bitcoin legal *(reversed coding)*; 3) There should be punishment for all Bitcoin providers; and 4) Government should buy a lot of Bitcoin *(reversed coding)*. Answering options to all questions range from 1 (fully disagree) to 7 (fully agree) plus a "don't know" option.

The following 3 statements and items are part of *the crypto ownership (past, current or expected) index*: 1) Current or past Crypto ownership; 2) The likelihood that the respondent will own Bitcoin 5 years from now; and 3) Preference of having €(or £)10,000 worth of crypto rather than savings, real estate, stocks, or gold. We recode these three variables as binary.

G Crypto ownership in the UK and the Netherlands since 2017

Figure G.1 shows crypto ownership over time in either country according to our data.

Figure G.1: Crypto ownership in the UK and the Netherlands, 2020-2023

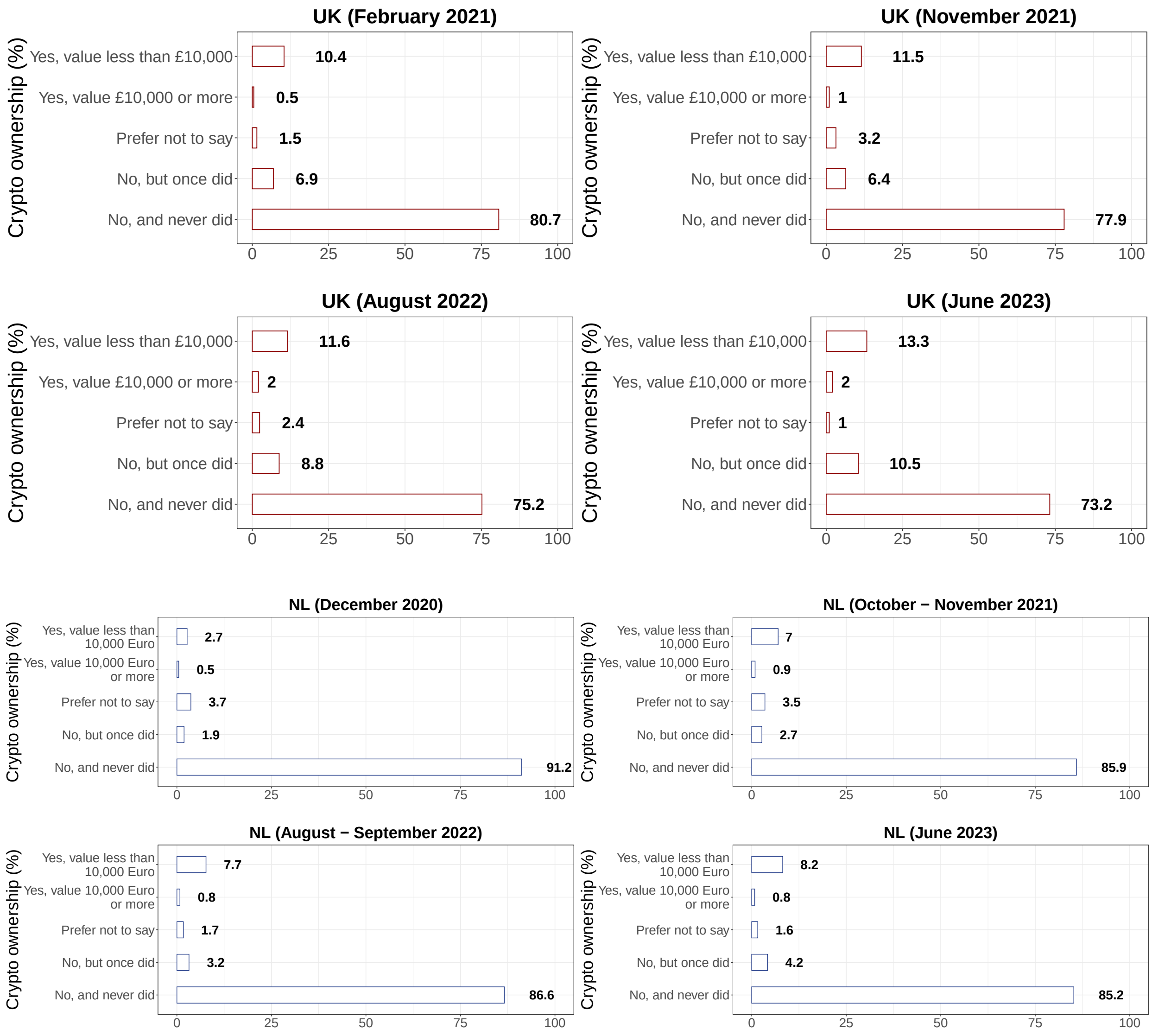

As Figure G.1 indicated, the proportion of those owning crypto has grown over time in both countries. In the UK, it evolved from a baseline level of 10.9% in February 2021 (wave 1) to 15.3% in June 2023. In the Netherlands, only 3.2% of the population owned crypto in December 2020 (wave 1). However, that proportion had almost tripled, to 9.0%, by June 2023 (wave 4).

The question of crypto ownership has been asked before. This allows us to benchmark our respondents' answers to the questions that make for our unique contribution to the body of knowledge. How do our crypto possession figures compare with those reported by other reputable research agencies? Figure G.2 shows ours (in red) along with all others we could find, adding up to a total of eight strings of data points.

As it turns out, our UK survey (commissioned to research firm Survation) consistently returns the highest estimates, see the upper panel of Figure G.2. At the same time, our Dutch survey (carried out by research agency Kantar) produces the lowest, see the lower panel. Despite the considerable differences in ownership level between our surveys, the actual level may be very similar across countries. In any case, adoption in these countries seems to have followed a similar trajectory so far, running from somewhere between 2% and 7% five years ago to somewhere between 8% and 15% now. This is important to bear in mind when discussing other demand-side observations in this paper, some of which may falsely suggest a wide gap between country-level adoption rates.

Figure G.2: Crypto ownership in the Netherlands and the UK, 2017-2023 (benchmarking)

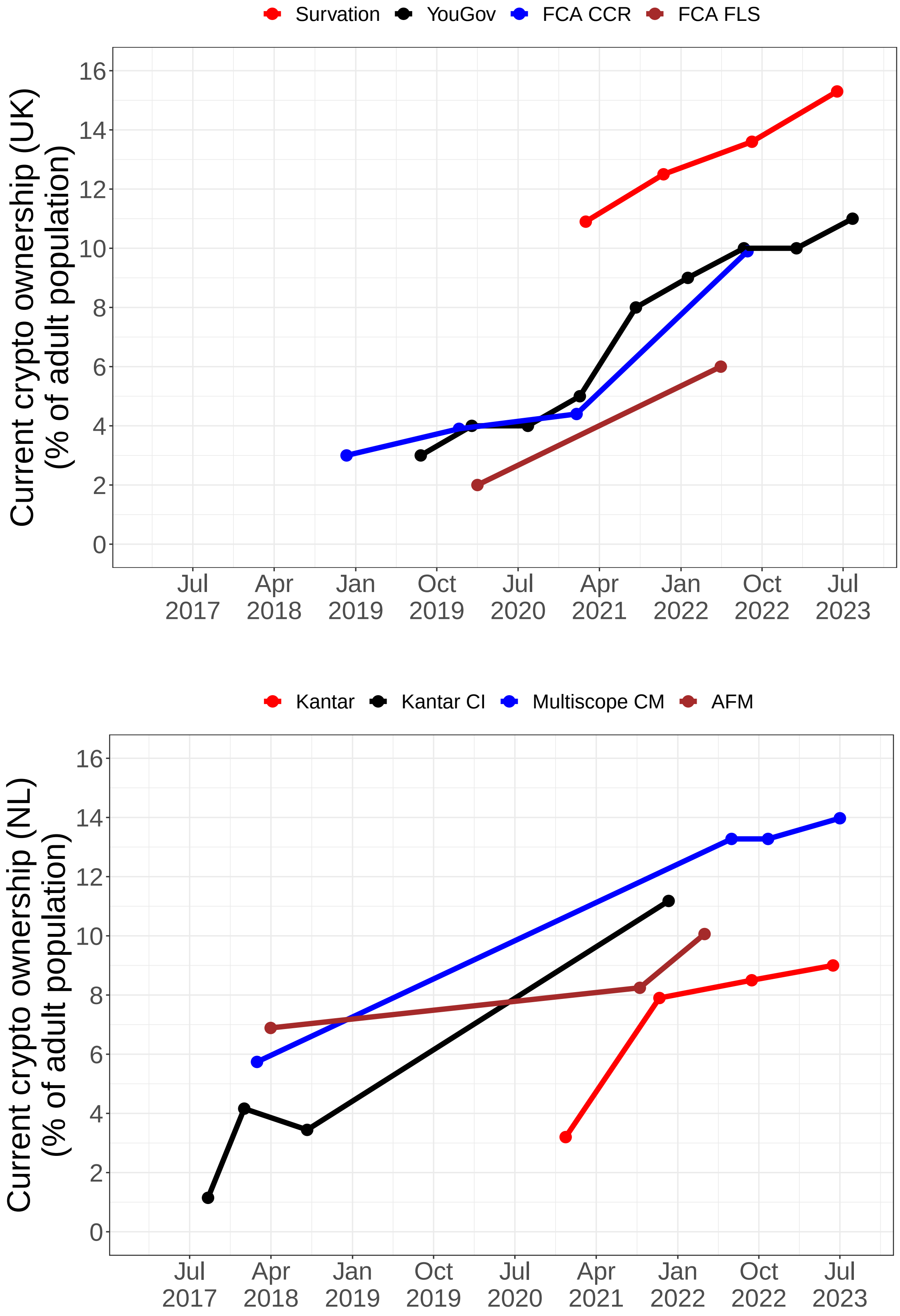

H Analysis of large crypto owners and Bitcoiners

For some parties, large bag holders may form a critical mass among their supporters in the near future.

Most crypto holders have a small amount, and many will have forgotten about it. Although prices have fluctuated, the median amount in the UK is stable at £100 worth of Bitcoin plus sometimes another sum of altcoins, the median of which varies between £37 in wave 4 to £100 in wave 3. In the Netherlands, the median varied from €225 in wave 3 to €750 in wave 1 for Bitcoin and from €60 in wave 1 to €600 in wave 2 for alts. It might make more sense to look at citizens who own a substantial amount for serious speculation or for substantial saving.

When examining citizens with a sizable crypto portfolio, numbers are small. This said, we have seen this group exploding in the UK (from 0.4% to 2.1% of all voters) while its Dutch counterpart almost doubled (from 0.5% to 0.9%). Note that these increases occurred notwithstanding a plummeting of crypto prices. For example, the share of UK citizens with a crypto stack worth more than £10,000 doubled between wave 2 and 3 even though Bitcoin fell from £51,000 to £17,000 during that period. Among these risk seekers, there is again a left-wing skew in Britain and an unclear picture in the Netherlands. Britons who have over £10,000 worth of crypto assets tend to vote left (N=66). The most preferred party, Labour, seems overrepresented (35% versus 23% in the entire sample) and easily beats the runners up, the underrepresented Tories (15% versus 32%). Turning to Dutch who hold over €10,000 in crypto, by contrast, we see a rightist skew (N=38). The VVD, in pole position, appears overrepresented (24% versus 15%), just as the PVV right behind it (16% versus 8%). However, these individuals are such tiny fraction of the Labour (1.6%), VVD (1.2%), and PVV (1.5%) electorate that they are unlikely to exert influence in this way. Their power over these parties might rather go through party finance.

Perhaps the most important voters here are those who feel strongly about the issue. Just as some conservative parties in Europe are currently held hostage by anti-immigration voters, some parties might in the future have a strong incentive to lend their ear to "Bitcoiners": Voters who are not only positive about Bitcoin but also attach much importance to it. To see which parties may have to deal

with this type of voter in the future, we select those voters who score higher than the midpoint on the positive Bitcoin factor and also higher than the midpoint on salience.[1] In the UK, Bitcoiners make up around 7% of LibDem, Tory, and SNP support, whereas Labour has a larger share of Bitcoiners (11.7%). Parties with more Bitcoiners than Labour are all to its left. Most importantly, no fewer than 25.5% of Green voters count as Bitcoiners. This suggests that some progressive leftist parties might soon come under pressure of a critical mass of Bitcoiners.[2] Numbers are tiny in the Netherlands, where none of the parties has over 6% of Bitcoiner supporters. This may be due to a potential underrepresentation of crypto holders in our Dutch sample (see Figure G.2 in Appendix G). Similarly, the high numbers for Green and Labour voters may result from the potential overrepresentation that the same Figure G.2 suggests. Still, it seems clear that the trends suggest that progressive parties may soon face a pro-Bitcoin bloc among their supporters.

---

[1]The question asked in wave 3 and 4 in each country is: "How important do you think the following issues are?" followed by five issue domains, presented in random order: "environmental issues (e.g., climate change)," "economic issues (e.g., cost of living)," "immigration issues (e.g., asylum seekers)," "foreign affair issues (e.g., help Ukraine)," and "financial innovation issues (e.g., Bitcoin)." Answering options varied from 1 ("completely unimportant") to 7 ("extremely important") with a "do not know" option. Those who indicated 5, 6, or 7 when asked about financial innovation and scored higher than the midpoint on the positive Bitcoin dimension (crypto positive impact index in both countries) were coded "1" and the rest was coded "0".

[2]The highest scores are among supporters of SDLP (N=15) and Plaid Cymru (N=25) but these are surrounded by great uncertainty due to the small numbers of observations. Bitcoiners are 6.8% of LibDems (N=444), 7.3% of Tories (N=1,627), 7.4% of SNP voters (N=148). The number of observations for Labour is N=1,183 and for the Greens N=111.

I Party position on Bitcoin and issue yield (full samples)

Figure I.1: Party position on Bitcoin and issue yield in the UK and the Netherlands (full samples)

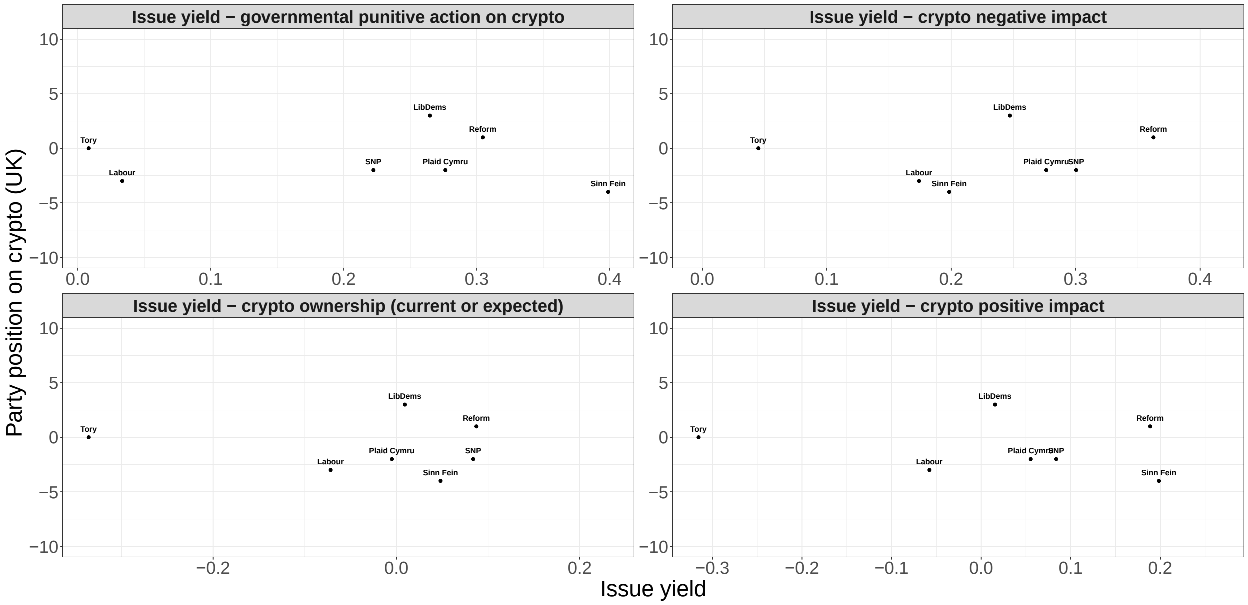

The plots do not include the parties with no position on crypto: Green Party, Alliance, SDLP and DUP.

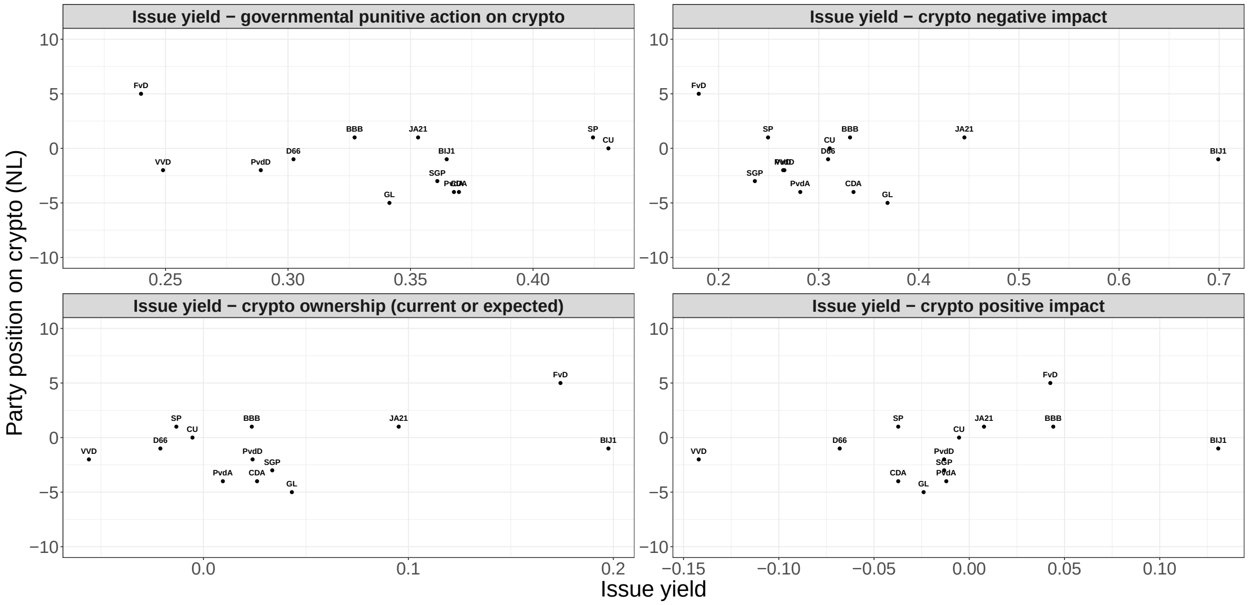

The plots do not include the parties with no position on crypto: PVV, Volt, and 50 PLUS.

Figure I.2: Party position on Bitcoin and issue yield in the UK and the Netherlands (full samples, parties with no positions coded as zero)

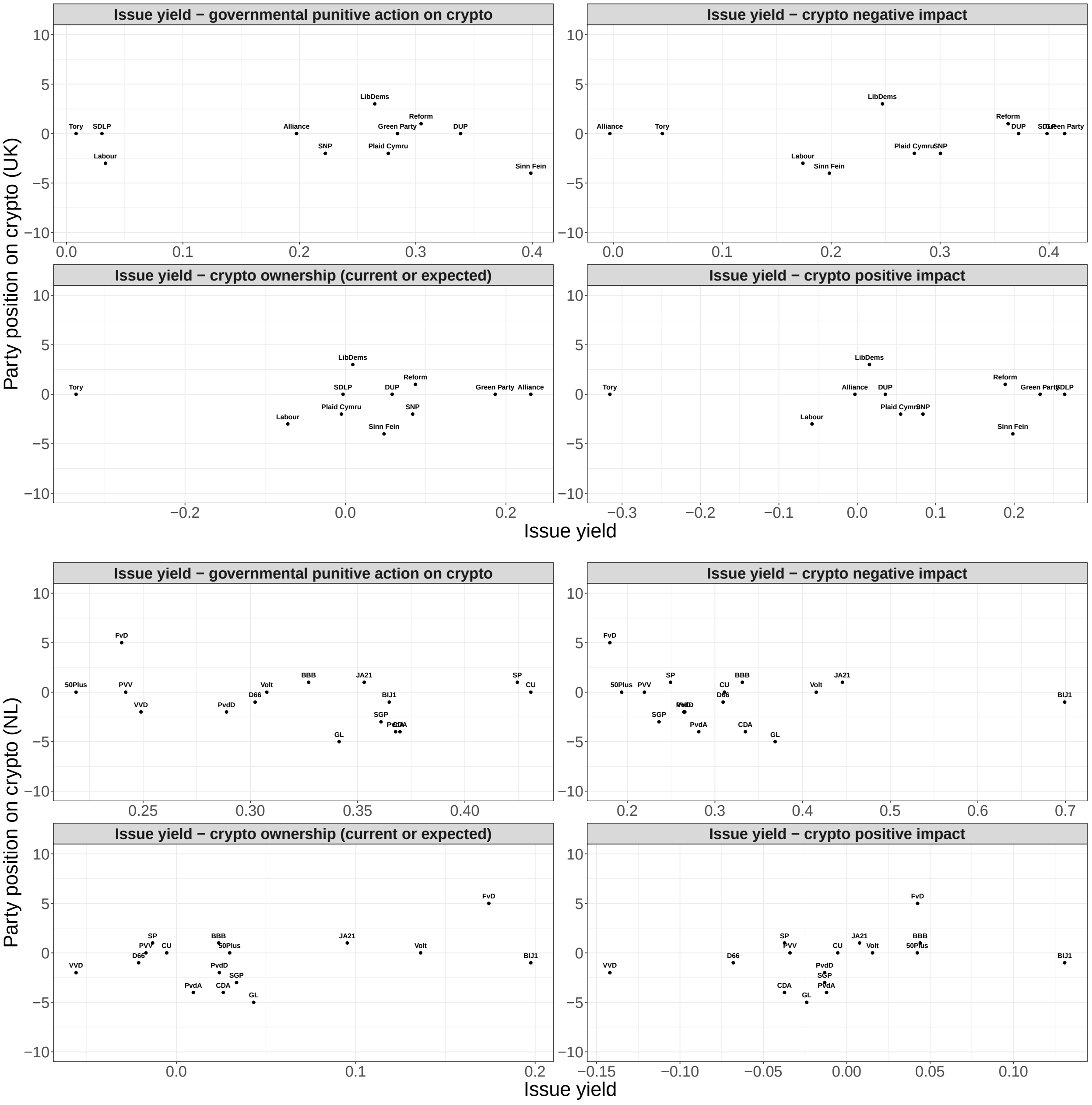

J Party position on Bitcoin and issue yield (the privileged groups)

Figure J.1: Party position on Bitcoin and issue yield in the UK and the Netherlands (the privileged groups)

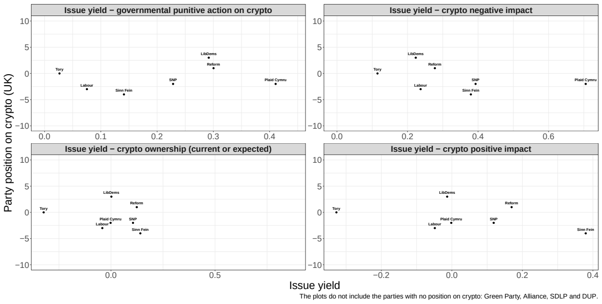

(a) Male (UK)

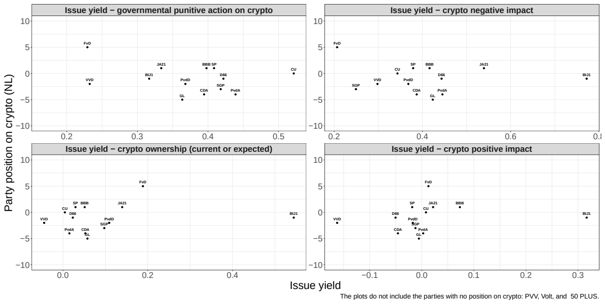

(b) Male (NL)

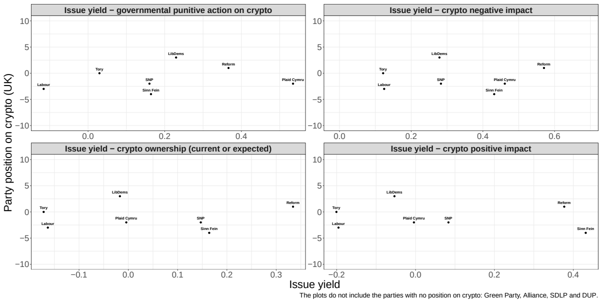

(c) High education (UK)

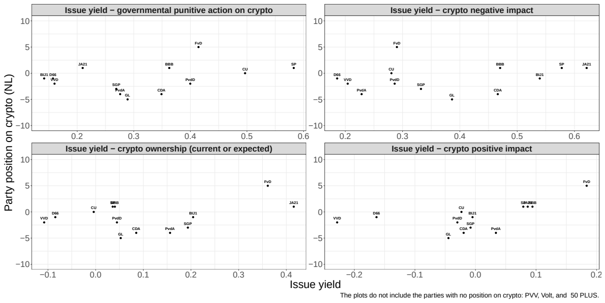

(d) High status (NL)

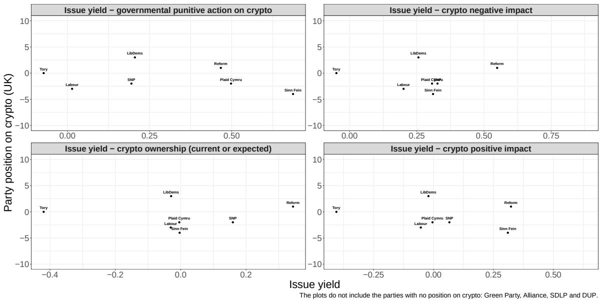

(e) High income (UK)

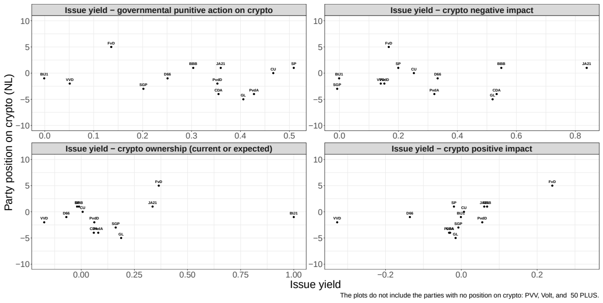

(f) High income (NL)

Figure J.2: Party position on Bitcoin and issue yield in the UK and the Netherlands (the privileged groups, parties with no position coded as zero)

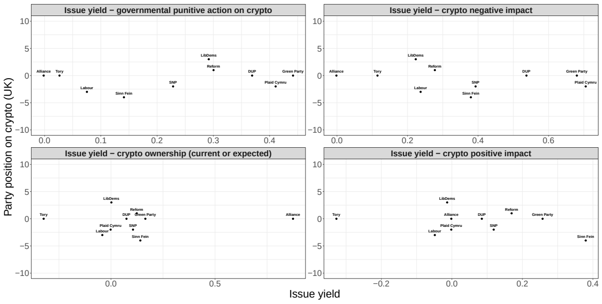

(a) Male (UK)

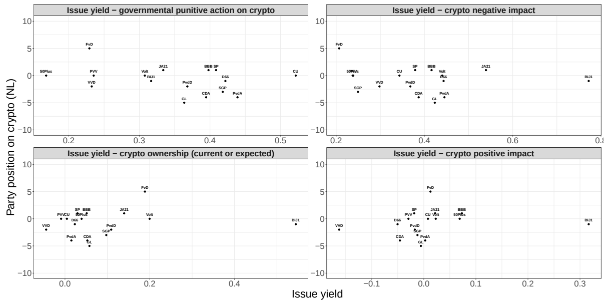

(b) Male (NL)

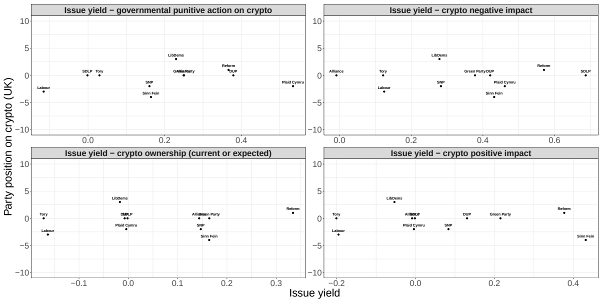

(c) High education (UK)

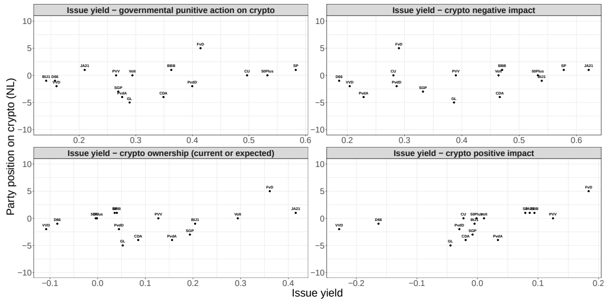

(d) High status (NL)

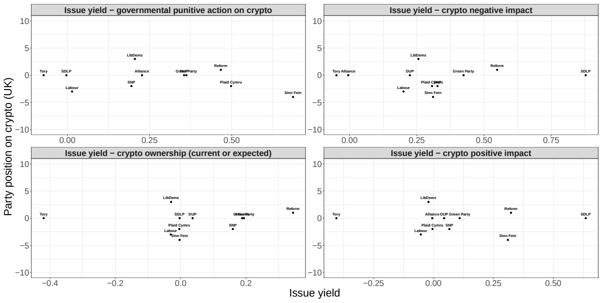

(e) High income (UK)

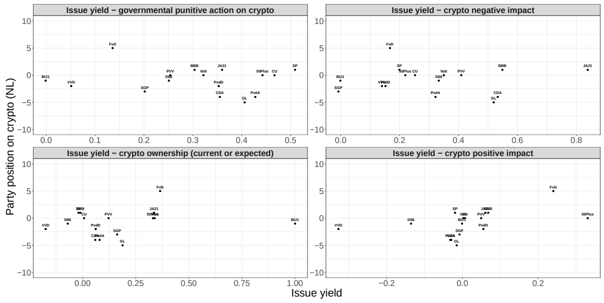

(f) High income (NL)

References

Béjaoui, Azza, Nidhal Mgadmi, Wajdi Moussa, and Tarek Sadraoui. 2021. "A short-and long-term analysis of the nexus between Bitcoin, social media and Covid-19 outbreak." *Heliyon* 7 (7).

Burnie, Andrew, and Emine Yilmaz. 2019. "An analysis of the change in discussions on social media with Bitcoin price." In *Proceedings of the 42nd International ACM SIGIR Conference on Research and Development in Information Retrieval*, 889–892.

Krippendorff, Klaus. 2018. *Content analysis: An introduction to its methodology*. Sage Publications.

Mai, Feng, Qing Bai, Jay Shan, Xin Shane Wang, and Roger Chiang. 2015. "The impacts of social media on Bitcoin performance."

Mai, Feng, Zhe Shan, Qing Bai, XinWang, and Roger Chiang. 2018. "How does social media impact Bitcoin value? A test of the silent majority hypothesis." *Journal of Management Information Systems* 35 (1): 19–52.

Matta, Martina, Ilaria Lunesu, Michele Marchesi, et al. 2015. "Bitcoin Spread Prediction Using Social and Web Search Media." In *UMAP workshops*, 1–10.

Philippas, Dionisis, Hatem Rjiba, Khaled Guesmi, and Stéphane Goutte. 2019. "Media attention and Bitcoin prices." *Finance Research Letters* 30:37–43.

Poongodi, M, Tu Nguyen, Mounir Hamdi, and Korhan Cengiz. 2021. "Global cryptocurrency trend prediction using social media." *Information Processing & Management* 58 (6): 102708.

Sylvester, Christine, Zachary Greene, and Benedikt Ebing. 2022. "ParlEE plenary speeches data set: Annotated full-text of 21.6 million sentence-level plenary speeches of eight EU states." *URL: https://doi. org/10.7910/DVN/ZY3RV7.*

Urquhart, Andrew. 2018. "What causes the attention of Bitcoin?" *Economics Letters* 166:40–44.
